# Gut‐derived *Lactobacillus* from exceptional responders mitigates chemoradiotherapy‐induced intestinal injury through methionine‐driven epigenetic modulation

**DOI:** 10.1002/imt2.70043

**Published:** 2025-05-14

**Authors:** Lu Yu, Zhenhui Chen, Shengqi Yin, Qiqing Guo, Yuchuan Chen, Jiaying Li, Yafang Wang, Xiangqiang Liu, Zi Xu, Yaowei Zhang, Yuqin Zhang, Zhihao Zheng, Keli Chen, Yanqing Ding, Hongying Fan, Zhifeng Liu, Yi Ding

**Affiliations:** ^1^ Department of Radiation Oncology, Nanfang Hospital Southern Medical University Guangzhou China; ^2^ Department of Microbiology, Guangdong Provincial Key Laboratory of Tropical Disease Research, School of Public Health Southern Medical University Guangzhou China; ^3^ State Key Laboratory of Organ Failure Research, Key Laboratory of Infectious Diseases Research in South China, Ministry of Education, Guangdong Provincial Key Laboratory of Viral Hepatitis Research, Guangdong Provincial Clinical Research Center for Viral Hepatitis, Department of Infectious Diseases, Nanfang Hospital Southern Medical University Guangzhou China; ^4^ Department of Radiotherapy General Hospital of Southern Theatre Command Guangzhou China; ^5^ Department of Gastroenterology General Hospital of Southern Theatre Command Guangzhou China; ^6^ HuiQiao Medical Center, Nanfang Hospital Southern Medical University Guangzhou China; ^7^ Guangdong Province Key Laboratory of Molecular Tumor Pathology Guangzhou China; ^8^ The Department of Critical Care Medicine General Hospital of Southern Theater Command of PLA Guangzhou China

**Keywords:** acute chemoradiotherapy‐induced intestinal injury, *Lactobacillus*, *Lacticaseibacillus rhamnosus* DY801, Lti cells, methionine metabolism, metB, SAM

## Abstract

Acute chemoradiotherapy‐induced intestinal injury (ACRIII) is a common and debilitating complication in patients with colorectal cancer, significantly impairing both quality of life and treatment outcomes. This study aimed to investigate the role of the gut microbiome in mitigating ACRIII. Through bioinformatics analysis of clinical fecal samples and fecal microbiota transplantation (FMT) experiments in mice, we identified a strong association between a high abundance of *Lactobacillus* species and the absence of ACRIII. From the fecal samples of rectal cancer patients who achieved complete remission without experiencing ACRIII during chemoradiotherapy, 10 novel *Lactobacillus* strains were isolated and characterized. Among these, *Lacticaseibacillus rhamnosus* DY801 exhibited a robust capacity to synthesize methionine through metB. This microbial methionine production modulated methionine metabolism in host gut lymphoid tissue inducer (Lti) cells, without diminishing the therapeutic efficacy of chemoradiotherapy. Supplementation with methionine increased intracellular levels of S‐adenosylmethionine and enhanced histone H3 lysine 4 trimethylation (H3K4me3) in Lti cells. These epigenetic modifications led to the suppression of pro‐inflammatory cytokines interleukin‐17A (IL‐17A) and interleukin‐22 (IL‐22), ultimately reducing ACRIII severity. Our findings suggest that specific *Lactobacillus* strains derived from patients with exceptional treatment responses may offer a novel therapeutic avenue for preventing or alleviating ACRIII. This microbiome‐based approach holds significant potential for improving patient outcomes and enhancing the tolerability of chemoradiotherapy in colorectal cancer.

## Highlights

- Exceptional patient‐derived *Lactobacillus* strains, notably *Lacticaseibacillus rhamnosus* DY801, effectively alleviate acute chemoradiotherapy‐induced intestinal injury (ACRIII).
- *Lacticaseibacillus rhamnosus* DY801 modulates host gut cysteine and methionine metabolism.
- Lti cells are crucial pro‐inflammatory mediators in the pathogenesis of ACRIII.
- Microbial‐derived methionine promotes histone methylation in Lti cells and reduces ACRIII‐associated inflammatory responses.

## Graphical abstract

Using metagenomic sequencing and bioinformatics analysis of fecal samples from rectal cancer patients and corresponding mouse models, our study identifies a strong association between *Lactobacillus* abundance and acute chemoradiotherapy‐induced intestinal injury (ACRIII). Elevated *Lactobacillus* levels before treatment were consistently linked to reduced intestinal damage. From patients who achieved complete tumor remission without developing ACRIII, we isolated 10 novel *Lactobacillus* strains. Among these, *Lacticaseibacillus rhamnosus* DY801 emerged as the most effective in mitigating ACRIII. Multi‐omics analysis revealed that DY801 modulates methionine metabolism in host lymphoid tissue inducer (Lti) cells, leading to increased histone H3 lysine 4 trimethylation (H3K4me3). This epigenetic modification suppresses the secretion of pro‐inflammatory cytokines IL‐17A and IL‐22, ultimately alleviating intestinal injury.

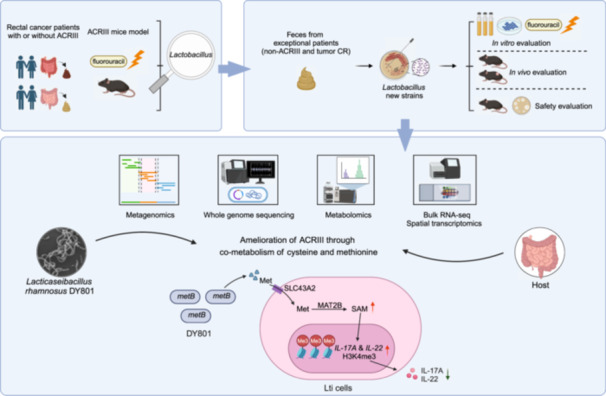

## INTRODUCTION

As neoadjuvant chemoradiotherapy remains the standard treatment for colorectal cancer, patient survival has notably improved, with some achieving complete clinical remission (CR) [1, 2]. Consequently, increasing attention has been given to the adverse effects of treatment [3, 4]. One of the most prevalent and distressing adverse effects of acute chemoradiotherapy is acute chemoradiotherapy‐induced intestinal injury (ACRIII), which typically manifests as diarrhea and abdominal discomfort in up to 80% of patients [3, 4]. Severe discomfort impairs the quality of life and interrupts cancer treatment, prolonging the overall treatment period [5, 6]. Chemotherapy, which is primarily based on fluorouracil or its prodrugs such as capecitabine (an oral fluoropyrimidine, that is, enzymatically converted into 5‐fluorouracil in the body), is a systemic treatment that causes damage to intestinal cells [7]. Although radiation is localized, it induces bystander effects, which can lead to intestinal injuries that extend beyond the treatment area [8]. Such injuries frequently result in intestinal stem cell apoptosis and other forms of cell death, compromising the intestinal barrier and allowing pathogenic bacteria to translocate and exacerbate inflammatory damage [9, 10, 11].

To date, no standardized effective treatment regimen exists for ACRIII [12]. Consequently, most medical centers rely on clinical experience to conduct trial‐and‐error treatment [12]. Previous studies have indicated that modulation of the gut microbiome through fecal microbiota transplantation (FMT), probiotics, and prebiotics can mitigate intestinal injuries by inhibiting excessive immune activation [13, 14, 15]. Nevertheless, FMT is challenging, including selection of donors and recipients, preparation of microbiota solutions, choice of transplantation routes, and safety concerns [13, 16]. The efficacy of probiotics derived from healthy individuals varies among studies [14, 15].

This study aimed to identify a novel microbiome‐based approach to treat ACRIII. Through metagenomic analysis of the intestinal microbiome and construction of a Random Forest deep‐learning model, we identified a high abundance of *Lactobacillus spp*. in the gut before treatment as a key factor in avoiding ACRIII. In clinical practice, we observed a subset of exceptional patients who, despite their cancer diagnoses, did not develop ACRIII throughout neoadjuvant therapy and achieved clinical and pathological CR. The intestinal microbiome serves as a natural reservoir for suitable *Lactobacillus* spp. Based on this insight, 10 wild *Lactobacillus* strains were isolated and subjected to comprehensive in vitro and in vivo screening. Through a comprehensive screening process, we identified *Lacticaseibacillus rhamnosus* (*L. rhamnosus*) DY801 as a promising candidate for treating ACRIII. This strain produces methionine through the *metB* gene, which modulates histone H3 lysine 4 trimethylation (H3K4me3) in host gut lymphoid tissue inducer (Lti) cell, thereby suppressing interleukin‐17A (IL‐17A) and interleukin‐22 (IL‐22) secretion.

## RESULTS

### High abundance of *Lactobacillus* in the gut correlates with the absence of ACRIII in patients

**Figure 1:**
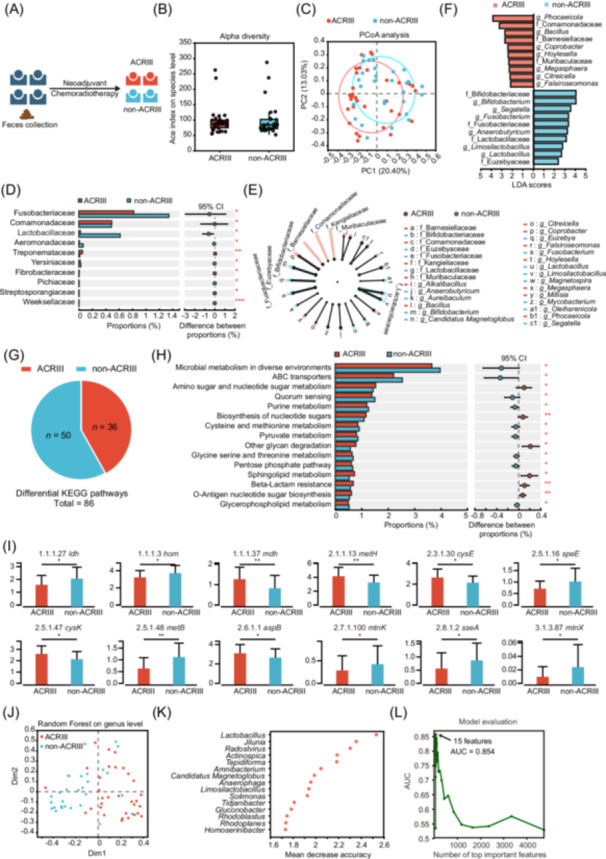
Fecal metagenomics links pretreatment gut *Lactobacillus* depletion to acute chemoradiotherapy‐induced intestinal injury (ACRIII). (A) Workflow of clinical data analysis. (B) Box plot comparing the difference in alpha diversity (Ace) between ACRIII and non‐ACRIII groups. (C) Principal coordinate analysis (PCoA) plot showing the difference in beta diversity between ACRIII and without ACRIII. (D) Bar chart showing the differential bacterial families between ACRIII and without ACRIII. (E) Cladogram showing the phylogenetic relationships of bacterial taxa found to be significantly associated with ACRIII or non‐ACRIII patients by linear discriminant analysis effect size (LEfSe). (F) The bar chart shows the linear discriminant analysis (LDA) scores. (G) Pie chart showing the number of differentially enriched ‌Kyoto Encyclopedia of Genes and Genomes (KEGG) pathways between ACRIII and without ACRIII. (H) Bar chart showing the top 15 proportions of differential KEGG pathways between ACRIII and non‐ACRIII patients. (I) Bar charts showing enzymes exhibiting statistically significant differences in the cysteine and methionine metabolism pathways. (J) Scatter plot showing sample distribution and classification results based on a Random Forest classifier at the genus level. (K) Dot plot showing the mean decrease accuracy for assessing the importance of each bacterial genus feature. (L) Line chart showing area under the curve (AUC) values when incorporating different numbers of the most important bacterial genus features. Significance: *p < 0.05, **p < 0.01, and ***p < 0.001; data represent two or three independent experiments. AUC, area under the curve; CI, confidence interval; PCoA, principal coordinate analysis.

ACRIII is common in patients with colorectal cancer, and the gut microbiota may play a significant role in this process. To investigate this, we collected baseline fecal samples and clinical characteristics from 61 patients with rectal cancer undergoing neoadjuvant chemoradiotherapy, and conducted metagenomic sequencing to dynamically monitor ACRIII occurrence throughout the treatment process (Figure 1A and Tables S1, S2).

The analysis of species‐level alpha diversity revealed no statistically significant intergroup differences (Figure 1B, *p* = 0.5335), but beta diversity analysis revealed distinct clustering (PC1: 20.40%, PC2: 13.03%; Figure 1C). Posttreatment non‐ACRIII patients exhibited higher relative abundances of *Fusobacteriaceae* and *Lactobacillaceae*, whereas ACRIII patients were enriched in *Comamonadaceae*, *Treponemataceae*, and *Streptosporangiaceae* (Figure 1D). Moreover, LefSe analysis and linear discriminant analysis (LDA) score visualization confirmed elevated *Bifidobacteriaceae*, *Lactobacillaceae*, *Bifidobacterium*, *Limosilactobacillus*, and *Lactobacillus* in non‐ACRIII patients (Figure 1E,F).

Differential Kyoto Encyclopedia of Genes and Genomes (KEGG) pathway analysis of metagenomic data identified 86 KEGG pathways, including 36 significantly enriched in ACRIII patients and 50 in non‐ACRIII patients (Figure 1G and Table S1). The top 15 pathways were selected for further analyses. Amino sugar and nucleotide sugar metabolism, and nucleotide sugar biosynthesis pathways were predominantly enriched in ACRIII patients, whereas microbial metabolism in diverse environments, purine metabolism, and cysteine and methionine metabolism were mainly enriched in non‐ACRIII patients (Figure 1H). The enzymes exhibiting statistically significant discrepancies in the cysteine and methionine metabolic pathways through metagenomics were presented in Figure 1I.

A Random Forest machine learning algorithm was used to identify key microbial features associated with ACRIII. The distribution of two sample groups are illustrated in Figure 1J, demonstrating notable differences. Using mean decrease in accuracy, *Lactobacillus* was identified as the most important genus, with *Limosilactobacillus* ranking ninth (Figure 1K). The construction of models incorporating varying numbers of the most important features revealed that the incorporation of 15 features yielded the highest area under curve (AUC, AUC = 0.854) (Figure 1L and Table S2). These findings indicate that patients with high abundance of *Lactobacillus* and *Bifidobacterium* were less likely to experience ACRIII, and *Lactobacillus* was identified as a highly significant feature.

To further elucidate the relationship between gut microbiota and ACRIII, we used a mouse model of FMT (Figure S1A). Following a single fraction of whole‐abdomen irradiation and three intraperitoneal injections of 5‐fluorouracil, the model mice exhibited significant diarrheal symptoms beginning on Day 5 (Figure S1B). Statistical analysis of the fecal samples revealed a notable reduction in fecal output in the RCT group, while FMT demonstrated a substantial improvement in diarrhea symptoms, although it did not fully restore the condition to that of the Control group (Figure S1C). Dynamic monitoring of mouse weight and survival demonstrated that mice subjected to RCT exhibited severe weight loss and required euthanasia by Days 5 and 6 due to acute diarrhea, resulting in a mortality rate of 100% (Figure S1D,E). In contrast, the mice that received FMT exhibited notable improvements in weight loss and reduced mortality rates (Figure S1D,E). Assessment of intestinal permeability using fluorescein isothiocyanate–labeled dextran (FITC‐dextran) revealed an increase in permeability in the RCT group, which was partially mitigated by FMT (Figure S1F).

Given the severe diarrhea observed between Days 5–6, the mice were euthanized on Day 6 for histopathological analysis of various intestinal segments (Figure S1G). Statistical evaluation of villus length in the small intestine revealed that RCT significantly reduced the length of jejunal villi, whereas FMT only partially restored this damage (Figure S1H). Furthermore, RCT markedly decreased ileal villus length, whereas FMT completely restored this injury (Figure S1I).

Cecal contents were collected from the mice for metagenomic analysis (Figure S1A). The analysis of species diversity revealed that the alpha diversity in the RCT group was lower than that in both the Control and FMT groups (Figure S1J). Beta diversity analysis indicated that PC1 accounted for 91.54% and PC2 for 4.32% of the variation, respectively (Figure S1K). A comparative analysis of the three groups revealed a significant reduction in the relative abundances of *Bacteroides*, *Lactobacillus*, and *Bifidobacterium* in the RCT group, with significant increases in these taxa after FMT (Figure S1L). A pronounced alteration in the abundance of *Akkermansia* was observed in the RCT group, which exhibited a notable decline after the FMT (Figure S1L).

These findings indicate a potential correlation between gut microbiota, including *Lactobacillus*, and ACRIII development. Although FMT has been demonstrated to partially alleviate ACRIII by modulating of the gut microbiome, further investigation is required to identify more effective and clearly defined microbiome modulation strategies.

### Isolation and screening of gut‐derived *Lactobacillus* from exceptional rectal cancer patient cohorts

Given the intricate composition of FMT, which presents a challenge for standardization and large‐scale production, and given that *Lactobacillus* was identified as a crucial feature in the Random Forest model, our investigation focused on *Lactobacillus*. Prior clinical studies that sought to improve radiation‐ and chemotherapy‐induced intestinal injury using commercially available or American Type Culture Collection (ATCC) standard *Lactobacillus* strains demonstrated limited efficacy. Therefore, we sought to adopt a novel approach to identify breakthroughs based on clinical observations.

**Figure 2:**
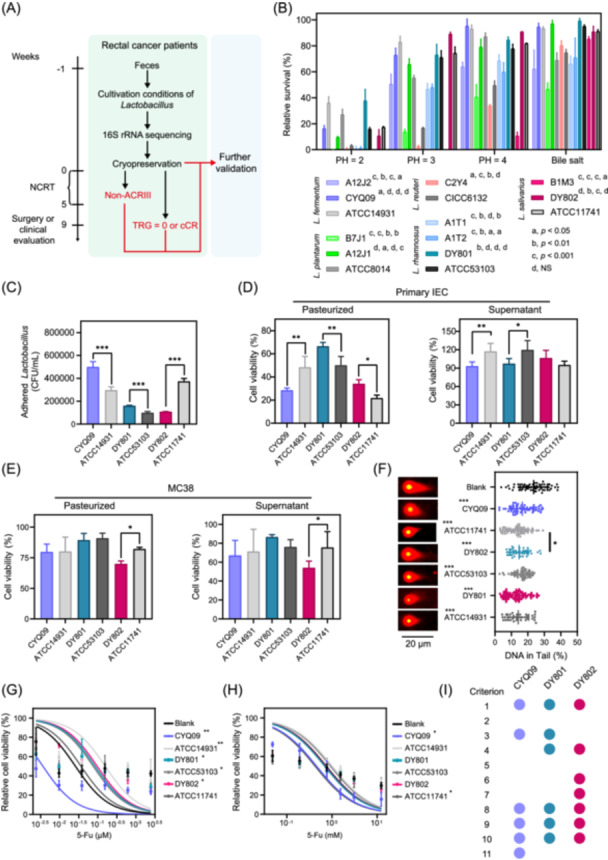
Isolation and screening of *Lactobacillus* strains from patients with rectal cancer in vitro. (A) Schematic representation of the isolation process of *Lactobacillus* strains derived from patients with rectal cancer. (B) Bar charts showing the survival rates of different *Lactobacillus* isolates and American Type Culture Collection (ATCC) strains in artificial gastric fluid at various pH levels, and in artificial intestinal fluid. Labels in the upper right corner of the strain represent the statistical results of survival rates at pH 2, 3, and 4 in artificial gastric fluid and artificial intestine compared to ATCC strains. (C) Bar charts showing colony‐forming unit (CFU) of different *Lactobacillus* strains adhering to primary intestinal epithelial cells. (D) Bar charts show the effects of heat‐pasteurized bacteria and supernatants from *Lactobacillus* strains on intestinal epithelial cell survival. (E) Bar charts show the effects of heat‐pasteurized bacteria and bacterial supernatants from various *Lactobacillus* strains on the survival rate of MC38 cells. (F) Representative comet assay images and dot plots showing the DNA percentages in the comet tails of irradiated primary intestinal epithelial cells after gel electrophoresis treated with bacterial supernatants from different *Lactobacillus* strains. (G) Fitted curves showing IC_50_ values of fluorouracil in primary intestinal epithelial cells treated with bacterial supernatants from various *Lactobacillus* strains. (H) Fitted curves demonstrating the IC_50_ values of fluorouracil in MC38 cells treated with bacterial supernatants from *Lactobacillus* strains. (I) Dot plot presenting the scoring of CYQ09, DY801, and DY802 strains based on the above criteria, where dots represent the scores obtained for each criterion. Significance: NS, *p* > 0.05, *p < 0.05, **p < 0.01, and ***p < 0.001; data represent two or three independent experiments. cCR, clinical complete remission; CFU, colony‐forming unit; IEC, intestinal epithelial cells; NCRT, neoadjuvant chemoradiotherapy; TRG, tumor regression grade; 5‐Fu, 5‐fluorouracil.

We identified a small subset of patients who exhibited a distinctive response during tumor treatment, particularly those with rectal cancer. Notwithstanding their cancer diagnosis, these patients did not develop ACRIII during neoadjuvant chemoradiotherapy and achieved CR, either clinically (cCR) or pathologically (pCR) (Figure 2A). Baseline fecal samples were collected before treatment, conditional cultivation of *Lactobacillus* was performed, and the identified bacterial strains were cryopreserved. If a patient was classified as exceptional, patient‐derived wild‐type *Lactobacillus* strains were revived for subsequent in vitro and in vivo screening (Figure 2A).

Ten strains with stable genetic characteristics were successfully isolated (Figure 2B). Acid and bile salt resistance assays showed these strains exhibited superior acid resistance and bile salt tolerance compared to ATCC strains. Strain DY801 demonstrated the highest survival rate (approximately 38%) at pH 2 (Figure 2B, Criterion 1). Although all isolated strains exhibited high bile salt tolerance (>90%), no statistically significant difference was observed when compared to the ATCC controls (Figure 2B, Criterion 2).

Primary intestinal epithelial cells were isolated and identified using cytokeratin 18 (CK‐18) staining (Figure S2A). The results of the adhesion assays demonstrated that CYQ09 and DY801 exhibited a greater capacity for adhesion to primary intestinal epithelial cells than the ATCC control strains (Figure 2C, Criterion 3).

Coculturing inactivated bacteria and their supernatants with primary intestinal epithelial cells demonstrated that the inactivated DY801 and DY802 strains significantly enhanced cell viability compared to the ATCC strains (Figure 2D, left panel, Criterion 4). However, the bacterial supernatants did not significantly enhance cell viability (Figure 2D, right panel; Criterion 5). In coculture experiments with murine colon cancer cells (MC38), inactivated DY802 demonstrated a significantly greater inhibitory effect on tumor cell survival than the ATCC strains (Figure 2E, left panel, Criterion 6). Furthermore, only the supernatant from DY802 demonstrated a notable reduction in tumor cell viability compared to that of the ATCC strains (Figure 2E, right panel, Criterion 7). The supernatants from the other strains did not promote tumor survival (Figure 2E).

The comet assay results indicated that CYQ09, DY801, and DY802 significantly enhanced the radiation resistance of primary intestinal epithelial cells (Figure 2F, Criterion 8). Conversely, these strains reduced the radiation resistance of MC38 tumor cells, rendering them more sensitive to radiation (Figure S2B, C, Criterion 9).

IC_50_ assays revealed that CYQ09, DY801, and DY802 markedly enhanced the chemotherapy tolerance of primary intestinal epithelial cells (Figure 2G, Criterion 10). Only CYQ09 demonstrated the capacity to inhibit the chemotherapy tolerance in MC38 cells, sensitizing them to chemotherapy (Figure 2H, Criterion 11).

A comprehensive analysis of the screening criteria is presented in Figure 2I, which indicates that CYQ09 and DY801 each achieved six points, while DY802 achieved seven points. Wild‐type *Lactobacillus* strains (*Limosilactobacillus fermentum* CYQ09, *L. rhamnosus* DY801, and *Ligilactobacillus salivarius* DY802) showed robust overall performance.

The safety of these wild‐type strains is a critical consideration for their potential clinical application. The antimicrobial susceptibility profiles of the three *Lactobacillus* strains were evaluated using a radar chart, which showed that all the strains met the established guidelines and exhibited no signs of abnormal antibiotic resistance (Figure S2D). These strains exhibited resistance to the clinical drugs, loperamide and sulfasalazine, indicating their potential for future clinical combination therapies (Figure S2D).

The results of the acute toxicity study indicated that CYQ09, DY801, and DY802 did not significantly affect the body weight of the mice (Figure S3A). Examination of vital organs revealed no notable alterations (Figure S3B). Histological analysis of these vital tissues using hematoxylin and eosin staining confirmed that the wild‐type *Lactobacillus* strains did not induce pathological damage (Figure S3C). The calculated organ indices for each mouse group demonstrated no abnormal alterations (Figure S3D–H). Hematological assessments of the blood samples collected from each group revealed no indications of hematological toxicity (Figure S3I–R). Moreover, the analysis of renal and hepatic functions revealed the absence of renal toxicity (Figure S3S,T) or hepatic toxicity (Figure S3U–W). Collectively, these findings indicate that the three wild *Lactobacillus* strains exhibited excellent safety profiles.

### *L. rhamnosus* DY801 significantly ameliorates ACRIII without compromising tumor treatment efficacy

**Figure 3:**
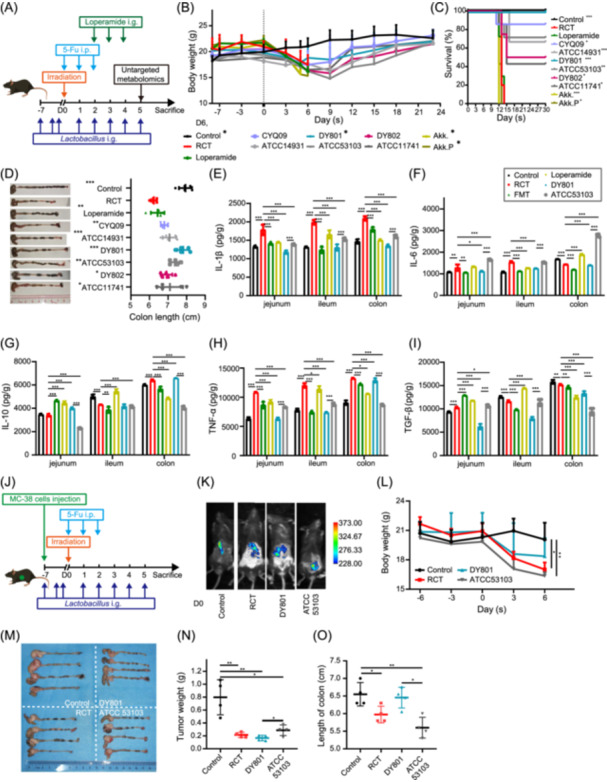
*Lactobacillus* strains derived from exceptional rectal cancer patients alleviate ACRIII without affecting tumor treatment efficacy in vivo. (A) Schematic representation of *Lactobacillus* monocolonization, *Akkermansia muciniphila* (AKK) monocolonization, ACRIII induction, loperamide treatment, and cecal content collection for untargeted metabolomics. (B) Body weight changes in mice after bacterial monocolonization, ACRIII induction, and loperamide treatment. *n* = 8 mice per group. Asterisks of group labels indicate statistical differences compared to the radiochemotherapy (RCT) group. (C) Survival rate in mice after different treatments. Asterisks of group labels indicate statistical differences compared to the RCT group. (D) Representative images and quantification of colon length. Asterisks in the upper left corner of the group labels indicate statistical differences compared to the RCT group. (E) The interleukin‐1 beta (IL‐1β) level in the jejunum, ileum, and colon of mice in each group. (F) The interleukin‐6 (IL‐6) level in each group. (G) The interleukin‐10 (IL‐10) level in each group. (H) The tumor necrosis factor‐alpha (TNF‐α) level in each group. (I) The transforming growth factor‐beta (TGF‐β) level in each group. (J) Schematic representation of *Lactobacillus* monocolonization, MC38 cell injection, and ACRIII induction. (K) The fluorescence intensity of orthotopic tumors in each group of mice was assessed using an in vivo imaging system on Day 0. (L) Body weight changes in mice after bacterial monocolonization, MC38 injection, and ACRIII induction. *n* = 4 mice per group. Statistical analysis of body weight on Day 6. (M) Representative images of tumors and the colon. (N) Dot plot showing the tumor weights of each group. (O) Dot plot showing colon length in each group. Significance: *p < 0.05, **p < 0.01, and ***p < 0.001; data represent two or three independent experiments. AKK.P, heat‐pasteurized Akkermansia muciniphila; i.g., intragastric; i.p., intraperitoneal.

Given the comparable in vitro screening and safety profiles exhibited by the three wild‐type *Lactobacillus* strains, we proceeded with in vivo experiments for further assessment. Before establishing the ACRIII model (Figure 3A), the mice were pre‐colonized by oral administration of *Lactobacillus* for 1 week. Continuous monitoring of mouse body weight and survival indicated that both the wild‐type and ATCC strains significantly mitigated weight loss and improved survival in diarrheal model mice. The DY801 strain achieved 100% survival, whereas CYQ09 strain achieved nearly 90% survival (Figure 3B,C). In contrast, the clinical drug loperamide demonstrated no significant effect on body weight or survival in the model mice, which aligns with the established efficacy of the drug in improving severe ACRIII during clinical treatment (Figure 3B,C). Notably, the administration of *Akkermansia muciniphila*, whether live or heat‐inactivated, reduced survival time in the model mice (Figure 3C). The colon length of each group was measured, and it was observed that mice treated with DY801 exhibited the longest colon length, indicating the most significant improvement in diarrhea‐induced colon contractions (Figure 3D).

To further investigate whether the colonization of DY801 is pivotal for its ameliorative effects on ACRIII, we conducted a rescue experiment using ampicillin, which targets *Lactobacillus* (Figure S4A). Dynamic monitoring of body weight and survival revealed that gavage with DY801 significantly improved both parameters in the mice. However, these beneficial effects were markedly reversed by antibiotic treatment (Figure S4B,C). Quantitative polymerase chain reaction (PCR) analysis of jejunal content on Day 6 posttreatment showed that antibiotic administration significantly reduced the abundance of colonized *L. rhamnosus*, indicating a decrease in intestinal colonization (Figure S4D). Histopathological examination of intestinal tissues via Hematoxylin and Eosin (HE) staining demonstrated that DY801 gavage significantly increased villus length and crypt depth while reducing the crypt‐to‐villus ratio in the jejunum. These morphological improvements were reversed by antibiotic treatment (Figure S4E–H).

We conducted animal experiments to investigate whether the combined administration of three *Lactobacillus* strains isolated from exceptional patients could enhance therapeutic efficacy (Figure S4I). Dynamic monitoring of body weight revealed that dual‐strain combinations containing DY801 (e.g., CYQ09 + DY801 and DY801 + DY802) significantly increased murine body weight by day 6 compared to the RCT group (Figure S4J). Although mixed *Lactobacillus* administration significantly improved survival in model mice, it remained inferior to single‐strain interventions (Figure S4K). To elucidate this discrepancy, we analyzed the intestinal contents at multiple time points to quantify the relative abundance of the *Lactobacillus* genus and its constituent species. The mixed‐strain gavage failed to enhance the abundance of the *Lactobacillus* genus compared to single‐strain administration, with potential antagonistic effects (Figure S4L). Furthermore, combining a single strain with one or two additional *Lactobacillus* strains did not significantly increase fecal abundance (Figure S4M–O). Collectively, these findings demonstrate that the combinatorial use of *Lactobacillus* strains from exceptional patients failed to produce synergistic effects (1 + 1 > 2).

Intestinal segments were collected from mice to assess the levels of inflammatory cytokines. The results demonstrated a notable elevation in the concentrations of interleukin‐1 beta (IL‐1β), interleukin‐6 (IL‐6), tumor necrosis factor‐alpha (TNF‐α), and transforming growth factor‐beta (TGF‐β) in the jejunum of the RCT group, whereas these cytokine levels exhibited a marked decline in the DY801‐treated group, with more pronounced reductions compared with ATCC 53103 (Figure 3E–I). Similarly, the ileum of the RCT group demonstrated elevated levels of IL‐1β, IL‐6, and TNF‐α, which were notably reduced following DY801 treatment, exceeding the reduction observed in the ATCC strain (Figure 3E–I). In the colon of the RCT group, the concentrations of IL‐1β, IL‐10, and TNF‐α were significantly increased, whereas DY801 treatment led to substantial decreases in IL‐1β and IL‐10 levels, again outperforming the ATCC strain (Figure 3E–I). Furthermore, FMT significantly suppressed the release of inflammatory cytokines in both the small and large intestines. However, loperamide was only effective in reducing IL‐1β and TNF‐α levels in the small and large intestines, respectively (Figure 3E–I). These findings indicate that *L. rhamnosus* DY801 significantly improved the intestinal inflammatory environment.

Blood samples were obtained from each group to analyze the inflammatory cytokine levels. The results demonstrated a notable elevation in the levels of IL‐1β, IL‐6, TNF‐α, and TGF‐β in the blood of the RCT group (Figure S5A–E). Following treatment with DY801, the levels of these cytokines significantly decreased, particularly the reduction in IL‐1β, which was more pronounced than that observed in the ATCC strain (Figure S5A–E). In addition, FMT markedly reduced the levels of IL‐1β and IL‐6 in the blood, whereas loperamide effectively inhibited IL‐1β, IL‐6, and TGF‐β (Figure S5A–E).

The effect of DY801 on the efficacy of tumor treatment was examined using a tumor‐bearing model. The mice were inoculated with mCherry fluorescent MC38 cells in the cecum to establish an in situ tumor model, as illustrated in Figure 3J. Before treatment, the tumor fluorescence intensity and area were comparable across all groups, with no significant differences observed (Figures 3K, S5F). Dynamic monitoring of body weight revealed that tumor chemoradiotherapy significantly decreased the weight of mice, whereas the DY801‐treated group exhibited a notable improvement in weight loss, significantly surpassing that of the ATCC strain (Figure 3L). Analysis of the in situ tumors and colon tissue collected from each group revealed a significant reduction in tumor weight in the RCT group compared with that in the Control group. Although the tumor weight in the DY801 group was slightly lower than that in the RCT group, the difference was not statistically significant. However, this was significantly lower than that in the ATCC 53103 group (Figure 3M,N). Statistical analysis of colon length revealed that administration of DY801 significantly alleviated colon contractions caused by radiotherapy and chemotherapy, with a pronounced effect compared to the ATCC strain (Figure 3O). In conclusion, DY801 effectively alleviated ACRIII without compromising the efficacy of in situ tumor treatment.

In addition, a subcutaneous tumor model was established (Figure S5G). Monitoring of mouse body weight demonstrated that the administration of tumor radiotherapy and chemotherapy caused a notable loss of weight, whereas the DY801‐treated group exhibited a marked recovery in weight, with effects that were significantly higher than those observed in the ATCC strain (Figure S5H). Subsequent analysis of the subcutaneous tumor volume indicated that the tumors in the RCT group were significantly smaller than those in the Control group. In addition, the data demonstrated that DY801 notably reduced both the volume and weight of subcutaneous tumors (Figure S5I–K). Therefore, DY801 effectively alleviated ACRIII and enhanced the efficacy of tumor treatment in the subcutaneous tumor model.

### Regulation of host intestinal cysteine and methionine metabolism by *metB* gene mitigates ACRIII

**Figure 4:**
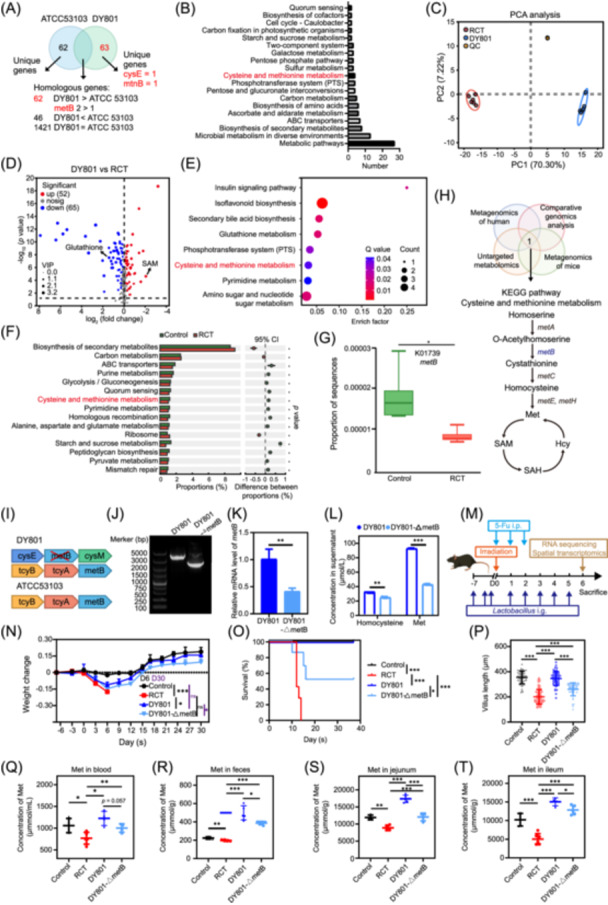
*Lacticaseibacillus rhamnosus* (*L. rhamnosus*) DY801 improves ACRIII activity by regulating cysteine and methionine metabolism. (A) Venn diagram showing the comparative genomic results between DY801 and ATCC 53103, where the symbols >, =, and < denote the comparison of homologous gene counts. *metB* 2 > 1 means that DY801 harbors two homologous *metB* genes, exceeding the single gene in ATCC 53103. (B) Bar plot showing KEGG pathway enrichment of differential genes between DY801 and ATCC 53103, with “Number” indicating the count of differential genes enriched in each pathway. All pathways exhibit *Q* values < 0.05. (C) Principal component analysis (PCA) plot showing metabolite differences between the RCT, DY801, and quality control groups. (D) Volcano plot showing differential metabolites between RCT and DY801 groups, with red and blue indicating upregulated and downregulated metabolites, respectively. All differential metabolites had variable importance in projection (VIP) scores ≥ 1, which were calculated using orthogonal partial least squares discriminant analysis (OPLSDA) analysis. (E) Bubble plot showing KEGG pathway enrichment of differential metabolites, with *Q* values < 0.05 and “count” indicating the number of differential metabolites enriched in each pathway. (F) Bar plots showing differential KEGG pathways between the Control and RCT groups, along with their proportions within the total pathways, and statistical significance. (G) Box plot uses the “Proportion of sequences” to display the abundance of *metB* genes in the Control and RCT groups. (H) Schematic of intersection analysis and key genes and metabolites within cysteine and methionine metabolic pathways. (I) Schematic representation of the location of *metB* in DY801 and ATCC 53103 cells. (J) Agarose gel electrophoresis confirmed the successful deletion of *metB* in DY801. (K) Bar charts showing qPCR analysis of *metB* transcriptional abundance in DY801 and ATCC 53103. (L) Bar plot showing liquid chromatography–mass spectrometry (LC‐MS) quantification of homocysteine and methionine in the supernatants harvested from DY801 and DY801‐△*metB* strains cultured in medium supplemented with 0.5% l‐cysteine for 24 h. Homocysteine and methionine abundances are indicated by the peak area in the mass spectrum. (M) Schematic representation of *Lactobacillus* monocolonization, ACRIII induction, and jejunal collection for bulk RNA sequencing and spatial transcriptomic analysis. (N) The Body weight changes of mice after bacterial monocolonization and ACRIII induction. *n* = 7 mice per group. Statistical analysis of body weight on Day 6. (O) The survival rates of mice in different groups. (P) Scatter plot showing the villus length of the jejunum in mice from each group. (Q) Scatter plot showing LC‐MS quantification of methionine in blood harvested from the mice in each group. (R) The LC‐MS quantification of methionine in feces. (S) The LC‐MS quantification of methionine in jejunum. (T) The LC‐MS quantification of methionine in ileum. Methionine abundance was indicated by the peak area in the mass spectrum. Significance: *p < 0.05, **p < 0.01, and ***p < 0.001; data represent two or three independent experiments. OPLSDA, orthogonal partial least squares discriminant analysis; PCA, principal component analysis; QC, quality control; no, no statistical significance; VIP, variable importance in projection.

Because of the remarkable in vitro and in vivo performance of DY801 compared to that of the ATCC strain, we comprehensively investigated the molecular mechanisms underlying its action. Comparative genomic analysis revealed that DY801 harbors 63 unique genes (Figure 4A). Furthermore, 62 homologous genes were identified in greater abundance in DY801 than in ATCC, whereas 46 homologous genes were present in lower abundance (Figure 4A). Enrichment analysis of the 125 genes unique to DY801 and those present in higher numbers than in the ATCC strain identified 19 enriched KEGG pathways, indicating a metabolic advantage at the gene level (Figure 4B).

Nontargeted metabolomic analysis of the cecal contents of mice revealed distinct metabolic profiles between the RCT and DY801 groups, with principal component 1 (PC1) accounting for 70.30% and PC2 accounting for 7.22% of the variance (Figure 4C). Differential analysis revealed that 52 metabolites were significantly elevated and 65 metabolites were significantly reduced in the DY801 group (Figure 4D). KEGG pathway enrichment of the 117 differentially expressed metabolites identified eight pathways, highlighting a metabolic advantage at the metabolite level (Figure 4E).

A reanalysis of the metagenomic data from the Control and RCT groups (Figure S1A) identified 15 KEGG pathways enriched in differentially expressed genes (DEGs), indicating that these pathways underwent significant alterations during ACRIII (Figure 4F). Intersection analysis of the pathways derived from the four analytical methods revealed a single common pathway, namely, cysteine and methionine metabolism. Furthermore, the key gene *metB* was significantly downregulated in the RCT group (Figure 4G,H). Among the distinctive genes in DY801 implicated in this pathway were *cysE* and *mtnB*, with *metB* exhibiting a homologous gene count of two, which was higher than that observed in the ATCC strain (Figure 4A). The metabolites regulated by DY801 in this pathway included glutathione and S‐adenosylmethionine (SAM) (Figure 4D). Therefore, it can be concluded that cysteine and methionine metabolism are crucial pathways for DY801 to exert its effects, with *metB* being a potential key gene.

Pan‐genomic analysis of *metB* was conducted on 1,520 human‐derived cultured gut bacteria. Among the top 10 genera with *metB*, *Bifidobacterium*, *Streptococcus*, and *Escherichia* were found to contain *metB* in nearly all cases, whereas 58% of *Lactobacillus* strains also harbored *metB* (Figure S6A). Analysis of genera containing a single *metB* gene revealed that *Bifidobacterium* constituted the largest proportion (41.47%; Figure S6B). Among those with two or more *metB* genes, *Lactobacillus* ranked second, accounting for 20.83% of the total (Figure S6C). These findings indicate that *Lactobacillus* and *Bifidobacterium* are the primary sources of *metB* in the human gut. We also analyzed the number of *metB* genes in other strains and found that CYQ09 and ATCC14931 contain two *metB* genes, whereas DY802 and ATCC11741 do not contain the *metB* gene.

To ascertain the role of *metB* in DY801's enhancement of ACRIII, we identified a distinctive *cysE*‐*metB*‐cysM gene cluster in DY801, which is involved in cysteine and methionine metabolism (Figure 4I). Owing to the lethal effects of a double knockout of *metB*, we constructed a *metB*‐knockout strain, designated DY801‐△metB (Figure 4I,J). Transcriptional validation demonstrated a notable decline in *metB* levels in the DY801‐△metB strain (Figure 4K). Mass spectrometry of the supernatants revealed significantly lower concentrations of homocysteine and methionine in the DY801‐△metB strain (Figure 4L). Similarly, the intracellular concentrations of homocysteine and methionine were significantly decreased in the knockout strain (Figure S6D).

*In vivo* studies revealed distinct temporal dynamics among groups: during the acute phase (Day 6 post‐model establishment), DY801‐treated mice exhibited intermediate body weights, which were significantly higher than the RCT group, although lower than the Control group, whereas DY801‐△metB mice showed comparable weights to the RCT group without statistical significance (Figure 4M,N). Extended observation over 1 month demonstrated progressive body weight recovery in the DY801 group reaching near‐Control group levels, whereas DY801‐△metB mice maintained significantly reduced body mass compared to their DY801 counterparts, accompanied by statistically divergent survival outcomes between these two experimental groups (Figure 4M‐O). These findings collectively demonstrate DY801's capacity for sustained therapeutic benefits through long‐term intervention. Histopathological analysis of the jejunum on Day 6 revealed shorter villi in the DY801‐△*metB* group compared to the DY801 group, whereas crypt lengths exhibited a similar trend (Figure 4P, Figure S6E,F). Statistical analysis of the crypt‐to‐villus length ratio revealed that the RCT group exhibited a significantly higher ratio than the Control and DY801 groups. In addition, the DY801‐△*metB* group demonstrated a significantly higher ratio than the DY801 group (Figure S6G).

Methionine levels in the blood, feces, and intestines of the mice were quantified. The concentration of methionine in the blood was markedly lower in the RCT group than that in the Control group. However, administration of DY801 led to a notable elevation in methionine levels. Knockout of *metB* in DY801 resulted in decreased methionine levels, which were still higher than those observed in the RCT group (Figure 4Q). Similar trends were observed in the fecal and intestinal segments (Figure 4R–T).

Our findings indicated that DY801 regulates methionine levels in the host intestine, thereby alleviating ACRIII. This effect depends on the presence of *metB*.

### Lti cells function as key pro‐inflammatory mediators in the progression of ACRIII

**Figure 5:**
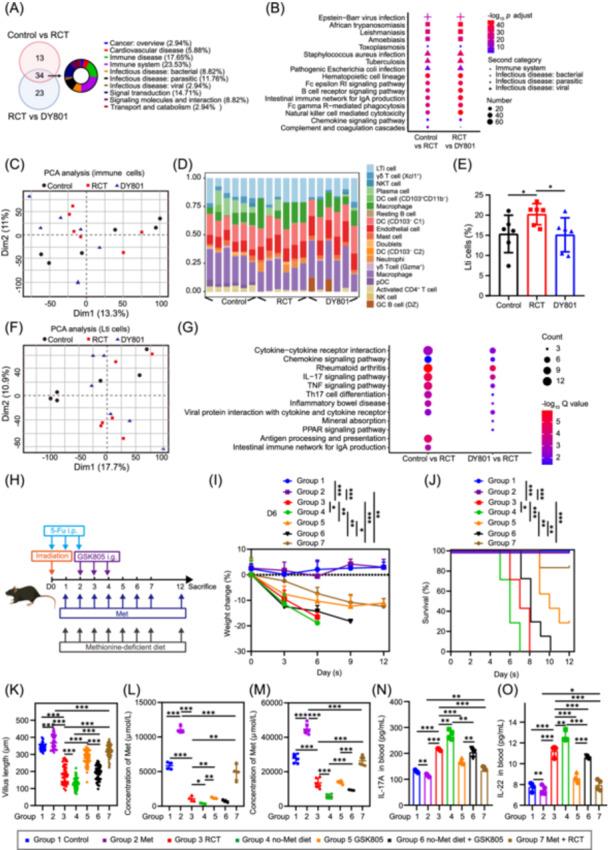
Lymphoid tissue inducer (Lti) cells are important pro‐inflammatory effector cells of ACRIII, and methionine inhibits Lti cells. (A) Venn diagram showing the number of KEGG pathways enriched with differentially expressed genes (DEGs) in the Control and RCT groups, as well as in the RCT and DY801 groups, along with the intersecting pathways. The proportion of the KEGG second category at the intersection is also presented. DEGs were defined as |log_2_ (fold change)| ≥ 2 and *p* ≤ 0.05. (B) Bubble plot showing intersecting pathways related to the immune system. (C) PCA plot showing gene differences of immune cells between the Control, RCT, and DY801 groups. (D) Stacked histogram showing the proportion of cells in each region of interest (ROI). Cell subtype annotation was performed using gene set variation analysis (GSVA) and GSE124880 (from GEO database). (E) Bar graph showing the proportion of Lti cells in each ROI. (F) PCA plot showing gene differences of Lti cells between the Control, RCT, and DY801 groups. (G) Bubble plot showing the TOP 10 KEGG pathways enriched with differentially DEGs in the Control and RCT groups, as well as in the RCT and DY801 groups in Lti cells. (H) Schematic representation of methionine supplementation in drinking water, feeding with a methionine‐deficient diet, GSK805 treatment, and ACRIII induction. Body weight changes (I) and survival rate (J) in mice after bacterial monocolonization and ACRIII induction. *n* = 6 mice per group. Statistical analysis of body weight on Day 6. (K) Scatter plot showing villus length in the jejunum of mice in each group. (L) Scatter plot showing LC‐MS quantification of methionine in blood harvested from mice in each group. Methionine abundance is indicated by the peak area in the mass spectrum. (M) LC‐MS quantification of methionine in jejunum. (N) Bar charts show interleukin‐17A (IL‐17A) level in the blood of the mice in each group. (O) Bar charts show interleukin‐22 (IL‐22) level in the blood of the mice in each group. Significance: *p < 0.05, **p < 0.01, and ***p < 0.001; data represent two or three independent experiments. GEO, Gene Expression Omnibus; GSVA, gene set variation analysis; Met, methionine; no‐Met diet, methionine‐deficient diet; ROI, region of interest.

Given that DY801 modulates host intestinal metabolism, we aimed to identify the key target cells responsible for its effects. Bulk RNA sequencing was performed on jejunal tissues from mice in the Control, RCT, and DY801 groups. Differential gene expression and KEGG pathway enrichment analyses were also performed. In total, 47 differentially enriched KEGG pathways were identified between the Control and RCT groups, whereas 57 differentially enriched pathways were identified between the RCT and DY801 groups. The intersection of these pathways included 34 that were primarily related to host immune responses, including the immune system and infectious diseases (Figure 5A,B and Table S3).

Based on these findings, we collected intestinal tissues from the three groups for spatial transcriptome analysis using CD45 to label immune cells within the intestine for sequencing. Quality controls demonstrated that the sequencing data were of high quality (Figures S6 and S7A,B). Principal component analysis (PCA) of the regions of interest (ROIs) demonstrated favorable intragroup consistency, with Dim1 accounting for 13.3% and Dim2 accounting for 11% (Figure 5C). The sequencing results for each ROI were annotated based on the published single‐cell data set GSE124880, and the proportions of different cell types were visualized (Figure 5D). Statistical analysis of cell type proportions revealed a significant increase in the number of Lti cells in the RCT group and a significant decrease in the DY801 group (Figure 5E). Similarly, γδT cells notably decreased in the RCT group and increased in the DY801 group (Figure S7C). The trend in the neutrophil proportion mirrored that of Lti cells (Figure S7D). The number of endothelial cells significantly increased in both the RCT and DY801 groups (Figure S7E). No statistically significant changes were observed in the other cell types. To further investigate the effects of DY801 and its metabolites on Lti cell functionality, we delineated new ROIs on the same slide based on the Lti cell characteristics. PCA demonstrated robust intragroup consistency (Dim1, 17.7%; Dim2, 10.9%; Figure 5F). DEG analysis revealed 210 DEGs between Control and RCT groups, and 42 DEGs between the DY801 and RCT groups (Figure S7F,G). KEGG enrichment identified eight shared pathways, including cytokine‐cytokine receptor interaction, chemokine signaling, IL‐17 signaling, TNF signaling, Th17 cell differentiation, and others (Figure 5G), indicating that DY801 and its metabolites affected both Lti cell quantity and function.

Given the high proportion of Lti cells among the statistically significant cell types, we selected them as the focal points for in vivo validation. Lti cells are characterized by retinoic acid‐related orphan receptor gamma (RORγ) expression, we administered the RORγ inhibitor GSK805 combined with a methionine‐deficient diet and oral methionine supplementation (Figure 5H). Mice fed a methionine‐deficient diet exhibited significantly reduced body weights and shorter lifespans compared to the RCT group (Figure 5I,J). Strikingly, these effects were reversed by GSK805‐mediated RORγ inhibition, restoring body weight and survival to levels comparable to the RCT group. Notably, methionine supplementation either alone or following RCT maintained or even enhanced body weight and survival relative to control and RCT groups, respectively (Figure 5I,J).

Histological analysis of jejunal tissues from mice on day 6 following hematoxylin‐eosin (HE) staining revealed that villus length in the GSK805 group was significantly longer than that in the RCT group, whereas villus length in the methionine‐deficient diet group was significantly shorter (Figures S7H, 5K). Villus length in the methionine‐deficient diet + GSK805 group was comparable to that of the RCT group, with no statistically significant difference (Figure 5K, *p* = 0.5822). A similar outcome was observed in crypt length analysis across the groups (Figure S7I). Statistical analysis of the crypt‐to‐villus length ratio demonstrated that the ratios in the GSK805 group were significantly lower than those in the RCT group, whereas the ratio in the no‐Met diet group was significantly higher. The ratio in the no‐Met diet + GSK805 group was comparable to that in the RCT group, with no significant difference (Figure S7J). Mice administered methionine supplementation alone exhibited significantly increased villus length and crypt depth relative to the Control group while maintaining comparable crypt‐to‐villus ratios between these groups. Post‐RCT methionine supplementation not only further enhanced both villus length and crypt depth measurements, but also significantly elevated the crypt‐to‐villus ratio compared to the RCT group (Figures S7H–J, 5K).

Methionine levels were quantified in the blood and jejunum of mice. Blood tests indicated that methionine levels were significantly reduced in the two groups that had been fed a methionine‐deficient diet but significantly increased in the group that received methionine supplementation (Figure 5L). Similar results were obtained for the jejunum (Figure 5M).

Lti cells are responsible for the production and secretion of IL‐17A and IL‐22, which play pivotal roles in regulating the intestinal mucosal barrier. Consequently, we quantified the levels of IL‐17A and IL‐22 in the blood of these mice. These findings demonstrate that the methionine‐deficient diet markedly elevated IL‐17A and IL‐22 levels in the blood, which were diminished by GSK805 administration and methionine supplementation. The combination of methionine‐deficient diet and GSK805 treatment resulted in IL‐17A and IL‐22 levels comparable to those observed in the RCT group (Figure 5N,O).

Similarly, we evaluated IL‐17A and IL‐22 levels in the blood of ACRIII mice treated with either DY801 or the DY801‐△metB strain, as previously described. Compared to the Control group, the RCT group exhibited markedly elevated levels of IL‐17A and IL‐22. Oral administration of DY801 resulted in a significant reduction in these levels, similar to those observed in the Control group. In contrast, oral administration of DY801‐△metB led to a partial reduction in levels, but these levels remained higher than those observed in the DY801 group (Figure S6H,I). Methionine supplementation alone moderately inhibited IL‐17A/IL‐22 levels compared to the Control group, whereas post‐RCT administration markedly suppressed their RCT‐induced upregulation (Figure 5N,O).

In conclusion, our results demonstrate that Lti cells play a pivotal role in the pathogenesis of ACRIII, and that DY801 and methionine can inhibit the production of IL‐17A and IL‐22.

### Increased methionine enhances SAM‐mediated H3K4 trimethylation, inhibiting Lti cell activation and cytokine secretion

**Figure 6:**
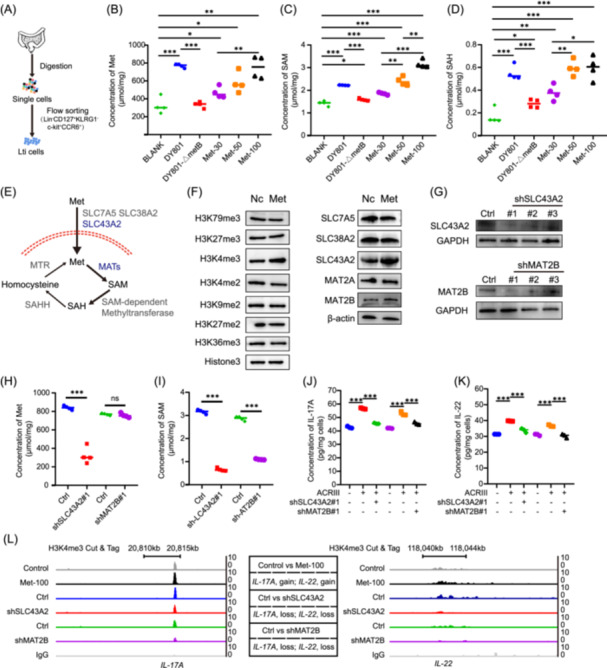
Methionine increases histone H3 lysine 4 trimethylation (H3K4me3) of *IL‐17A* and *IL‐22* in Lti cells and inhibits the release of pro‐inflammatory cytokines. (A) Flow cytometric analysis of Lti cells. (B) Scatter plots showing LC‐MS quantification of methionine in the culture medium of cells in each group, with the bacterial supernatant or varying concentrations of methionine added to the cell culture medium. (C) LC‐MS quantification of S‐adenosylmethionine (SAM) in the culture medium of cells. (D) LC‐MS quantification of SAH in the culture medium of cells. Methionine, SAM, and S‐adenosylhomocysteine (SAH) abundances are indicated by peak areas in the mass spectrum. (E) Schematic representation of key genes and metabolites involved in cellular methionine metabolism. (F) Western blot analysis images showing changes in the abundance of histone methylation modifications in the nucleus and alterations in the abundance of genes related to intracellular methionine metabolism after the addition of 100 mmol/L methionine to the Lti cell culture medium. (G) Western blot images demonstrating changes in the abundance of histone methylation‐ and methionine metabolism‐related genes in Lti cells after transfection with shRNA plasmids. (H) LC‐MS quantification of methionine in the culture medium of Lti cells after gene knockdown. (I) LC‐MS quantification of SAM in the culture medium of Lti cells after gene knockdown. (J) Bar charts show IL‐17A level in the cytoplasm of Lti cells following gene knockdown. (K) Bar charts show IL‐22 level in the cytoplasm of Lti cells following gene knockdown. (L) Assessment of *IL‐17A* and *IL‐22* binding at H3K4me3 loci in Lti cells using CUT&Tag sequencing. Significance: *p < 0.05, **p < 0.01, and ***p < 0.001; data represent two or three independent experiments. MATs, methionine adenosyl transferase; MTR, methionine synthase; SAH, S‐adenosylhomocysteine; SAHH, S‐adenosylhomocysteine hydrolase.

The effects of methionine on Lti cells were investigated by flow cytometry (Figure 6A). Cellular uptake of methionine is converted to SAM and S‐adenosylhomocysteine (SAH), which are essential for cellular methylation. Lti cells were established and cocultured with bacterial supernatants or with varying concentrations of methionine. Intracellular levels of methionine, SAM, and SAH were assessed. The results demonstrated that the addition of DY801 bacterial supernatant significantly increased the intracellular levels of methionine, SAM, and SAH, with an effect comparable to those observed with the addition of 100 µM methionine (Figure 6B–D). Similarly, the supernatant from DY801‐ΔmetB also demonstrated a significant increase in the intracellular levels of methionine, SAM, and SAH, albeit to a lesser extent than that observed for DY801 (Figure 6B–D). The increased methionine levels demonstrated a dose‐dependent effect on the intracellular levels of methionine, SAM, and SAH (Figure 6B–D).

The transport of methionine into cells depends on the activity of several membrane proteins, including solute carrier family 7 member 5 (SLC7A5), solute carrier family 38 member 2 (SLC38A2), and solute carrier family 43 member 2 (SLC43A2). Once inside the cell, methionine undergoes a series of transformations into SAM via multiple methionine adenosyl‐transferases (MATs), providing methyl donors for intracellular methylation reactions (Figure 6E). Following methionine treatment, the cells were collected for Western blot analysis, which revealed a significant increase in histone H3K4me3 in response to methionine addition to the culture medium. The protein expression of the methionine transporter SLC43A2 was significantly upregulated, accompanied by a marked increase in methionine adenosyltransferase 2B (MAT2B), the enzyme responsible for catalyzing the conversion of methionine to SAM (Figure 6F).

To further elucidate the roles of these genes, we used shRNA plasmids to knock down *SLC43A2* and *MAT2B* (Figure 6G). The knockdown of *SLC43A2* resulted in a notable decline in both intracellular methionine and SAM levels. Conversely, the knockdown of *MAT2B* did not affect methionine levels but led to a considerable reduction in SAM levels (Figure 6H,I). Moreover, the concentrations of IL‐17A and IL‐22 in the cell cytoplasm were evaluated, and a notable decrease in these cytokines was observed following the knockdown of either *SLC43A2* or *MAT2B* (Figure 6J,K).

Using Cut & Tag sequencing, we analyzed H3K4me3 levels within the cell nucleus and determined that methionine significantly enhanced H3K4me3 levels at the *IL‐17A* and *IL‐22* loci (Figures S8, 6L, *Q* value < 0.05, and |log_2_ (fold change)| > 1). Knockdown of either *SLC43A2* or *MAT2B* significantly reduced H3K4me3 levels at *IL‐17A* and *IL‐22* loci (Figure 6L, *Q* Value < 0.05, and |log_2_ (fold change)| > 1). Our findings indicated that methionine elevates H3K4me3 levels associated with IL‐17A and IL‐22 in Lti cells, ultimately inhibiting the transcription and secretion of downstream IL‐17A and IL‐22.

**Figure 7:**
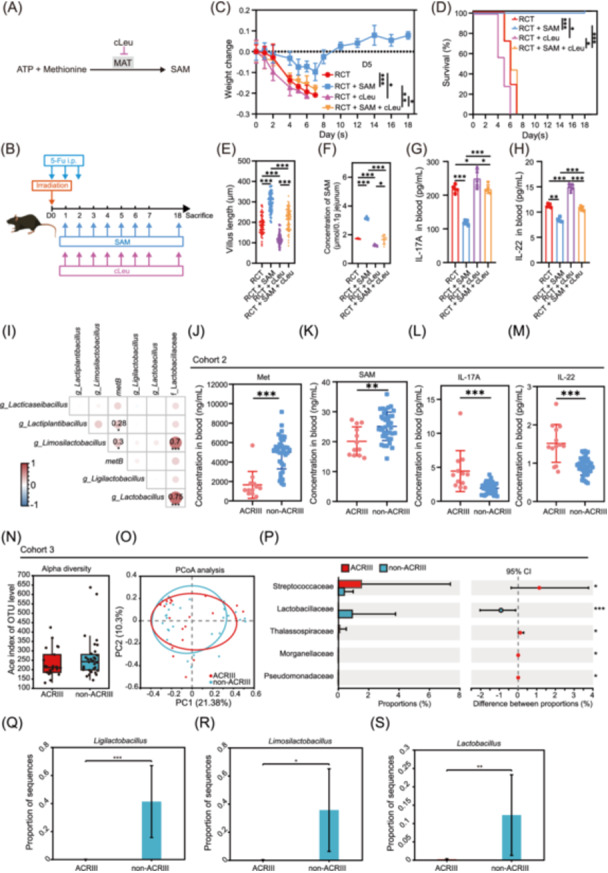
In vivo validation of methionine alleviates ACRIII. (A) Diagram showing actions of cLeu on the synthesis of SAM. (B) Schematic representation of cLeu and SAM treatment, ACRIII induction. (C) Body weight changes of mice after metabolites treatment and ACRIII induction. *n* = 7 mice per group. Statistical analysis of body weight on day 5. (D) Survival rates of mice in different groups. (E) Scatter plot showing the villus length of the jejunum in mice from each group. (F) Scatter plot showing LC‐MS quantification of SAM in jejunum harvested from the mice in each group. SAM abundance was indicated by the peak area in the mass spectrum. (G) Bar charts show IL‐17A level in the blood of the mice in each group. (H) Bar charts show IL‐22 level in the blood of the mice in each group. (I) Correlation plot showing the association between *metB* and the Lactobacillaceae family as well as the *Lactobacillus* genus in human feces metagenomics, where the size of the dots represents the magnitude of the correlation coefficient. (J) Scatter plot showing LC‐MS quantification of methionine in the blood of patients with and without ACRIII. (K) LC‐MS quantification of SAM in the blood of same patients. Methionine and SAM abundance were indicated by the peak area in the mass spectrum. (L) Scatter plot showing the concentration of IL‐17A in the blood of patients with and without ACRIII. (M) Scatter plot showing the concentration of IL‐22 in the blood of patients with and without ACRIII. (N) Box plot comparing the difference in alpha diversity (Ace) between ACRIII and non‐ACRIII patients. (O) PCoA plot showing the difference in beta diversity between ACRIII and without ACRIII. (P) Bar chart showing the differential bacterial families between ACRIII and without ACRIII. (Q) Scatter plot showing abundance of *Ligilactobacillus* in feces of patients after nCRT with and without ACRIII. (R) Scatter plot showing an abundance of *Limosilactobacillus* in feces of patients. (S) Scatter plot showing an abundance of *Lactobacillus* in feces of patients. Significance: *p < 0.05, **p < 0.01, and ***p < 0.001; data represent two or three independent experiments. ATP, adenosine triphosphate; cLeu, cycloleucine; OTU, operational taxonomic unit.

Subsequently, we conducted *an in vivo* validation using cLeu, a specific inhibitor of SAM‐mediated methylation (Figures 7A–H, S9). Dynamic monitoring of body weight and survival in mice revealed that SAM significantly improved survival and body weight in ACRIII model mice, whereas cLeu reversed the effects of SAM (Figure 7C,D). Histological examination and statistical analysis of jejunal tissues from each group indicated that SAM significantly increased villus length (Figures S9K, 7E) and crypt length (Figure S9L) and reduced the crypt‐to‐villus ratio (Figure S9M), confirming the dependency on SAM‐mediated methylation. Further, LC‐MS analysis of SAM levels in the jejunum showed a significant increase in SAM content following SAM treatment, which was markedly reduced by cLeu (Figure 7F). Analysis of blood samples for pro‐inflammatory cytokines revealed that SAM treatment significantly decreased the levels of IL‐17A and IL‐22, and these changes being reversed by cLeu treatment (Figure 7G,H). Under normal conditions, whereas SAM and cLeu treatments did not affect mouse survival and body weight (Figure S9B,C), SAM still significantly increased jejunal villus length and crypt length, reduced the crypt‐to‐villus ratio (Figure S9D–G), and significantly lowered IL‐17A and IL‐22 levels (Figure S9I,J, with a lesser reduction compared to the ACRIII model). These effects were reversed by cLeu treatment.

Our findings were translated into a clinical cohort from the initial study, focusing particularly on the abundance of the *metB* gene in patient fecal samples. The results of our analysis indicated that patients with ACRIII exhibited a lower abundance of *metB* in their fecal samples (Figure 1I). Correlation analysis between *metB* and *Lactobacillus* abundance revealed a significant positive relationship with the genera *Limosilactobacillus* and *Lactiplantibacillus* (Figure 7I). Furthermore, an additional cohort of LACR patients receiving nCRT was analyzed to investigate the relationship between blood levels of methionine, SAM, IL‐17A, and IL‐22. The results revealed that patients with ACRIII exhibited decreased methionine and SAM levels, along with elevated IL‐17A and IL‐22 levels, during treatment (Figure 7J–M). In the third cohort, fecal samples were collected from 57 patients with LARC before nCRT, and 16S rRNA sequencing was performed. The analysis of operational taxonomic unit (OTU)‐level alpha and beta diversity revealed no statistically significant differences in alpha and beta diversity between the groups (Figure 7N,O, *p* = 0.2604 and *p* = 0.7690). However, patients who developed ACRIII during nCRT exhibited significantly lower baseline levels of Lactobacillaceae, *Ligilactobacillus*, *Limosilactobacillus*, and *Lactobacillus* before treatment than those who did not develop ACRIII (Figure 7P–S). These results confirmed the previously observed association between low baseline fecal abundance of *Lactobacillus*, reduced blood levels of methionine and SAM, elevated IL‐17A and IL‐22, and the occurrence of ACRIII across the three independent clinical cohorts.

## DISCUSSION

The search for methods to predict the occurrence of ACRIII in patients with colorectal cancer or other abdominal and pelvic tumors during chemoradiotherapy presents a crucial medical challenge. Research in this field has affected patients' well‐being and treatment planning. In this study, we used baseline metagenomic data from patients before treatment to construct a deep‐learning model. This novel approach demonstrated a strong correlation between the composition of the gut microbiome before treatment, particularly the abundance of specific genera such as *Lactobacillus* and *Bifidobacterium*, and the development of ACRIII. *Lactobacillus* exhibited the highest importance score in our model, providing a valuable preliminary cohort and a scientific basis for the subsequent development of predictive models using multicenter big data. This will enable personalized predictions and early intervention.

In this study, the clinical cohort received the CapeOX regimen (capecitabine plus oxaliplatin), a standard neoadjuvant therapy for LARC, whereas the animal model used 5‐FU alone to induce intestinal injury. This difference does not compromise our conclusions, as capecitabine, an oral prodrug, is enzymatically converted into 5‐FU in its active form, ensuring comparable therapeutic effects. CapeOX was chosen for its clinical efficacy and tolerability, while 5‐FU was used in mice to align with established preclinical models [17, 18]. Both agents share a mechanism of action, thymidylate synthase inhibition, which supports the relevance of our findings. Future studies should explore capecitabine in animal models to further bridge preclinical and clinical insights.

Our study focuses on ACRIII rather than injuries caused by chemotherapy or radiotherapy alone, as it aligns with the clinical context of LARC, where nCRT is the standard of care according to the National Comprehensive Cancer Network (NCCN) and the European Society of Medical Oncology (ESMO) Guidelines [3, 5]. Although the synergistic benefits of combined chemotherapy and radiotherapy are well established, this multimodal approach also induces a unique spectrum of intestinal injuries distinct from those caused by either treatment alone [19]. By targeting ACRIII, we addressed a critical clinical challenge that significantly affects a patient's quality of life. This approach is supported by prior studies that have systematically investigated ACRIII as an integrated clinical entity, particularly exploring molecular mechanisms and therapeutic outcomes [3, 5, 19, 20].

In clinical practice, the prevention and treatment of ACRIII present several significant challenges [21]. Traditional drug treatments, such as loperamide, exhibit limited efficacy [21], a finding corroborated by our animal data. Furthermore, no clear indications exist for the use of prophylactic drugs. The use of FMT is limited to cases of severe or hemorrhagic diarrhea, which represents a significant limitation in terms of offering a proactive solution [22]. Moreover, the experimental results confirmed that FMT only partially alleviated weight loss, intestinal injury, and improved survival in mice. Despite the routine consumption of probiotics as dietary supplements, probiotics derived from healthy individuals have demonstrated limited efficacy in ameliorating ACRIII [14, 15, 23]. To address this situation, we isolated and screened multiple *Lactobacillus* strains with the potential to improve ACRIII from a cohort of exceptional patients with natural favorable outcomes, including *Limosilactobacillus fermentum* CYQ09, *L. rhamnosus* DY801, and *Ligilactobacillus salivarius* DY802, with DY801 demonstrating remarkable performance. This finding not only provides robust empirical support for exceptional patient‐derived probiotic strategies to prevent and treat ACRIII but also indicates the potential for subsequent isolation and screening of more suitable wild strains. Furthermore, the discovery of these probiotics provides new avenues for treating other inflammatory bowel diseases and necrotizing enterocolitis, underscoring their broad clinical applicability.

In preliminary trials with the well‐established probiotic *A. muciniphila* (AKK), neither live nor heat‐inactivated AKK exacerbated ACRIII, in stark contrast to its reported alleviating effects on other intestinal inflammatory conditions [24]. In another study, this phenomenon was attributed to the substantial uptake of mucins by AKK following radiotherapy. These mucins are used for energy production, and therefore compromises the integrity of the intestinal barrier. These findings provide crucial insights into the selection and application of probiotics.

Regarding the molecular mechanisms of DY801, our findings indicate that *metB* results in elevated methionine production, and this regulates activated Lti cells. These cells represent a crucial pro‐inflammatory cell type that has not been reported associated with ACRIII in the host gut. DY801‐derived methionine is taken up via SLC43A2 and converted to SAM by MAT2B, regulating Lti cells. This process increases H3K4me3 levels at the *IL‐17A* and *IL‐22* gene loci, directly suppressing their transcription and reducing cytokine secretion. Previous studies have shown that methionine enters T cells through SLC43A2 and is converted into SAM by MATs, regulating intracellular histone methylation (e.g., H3K79me2) [25, 26]. Our results align with these reports, demonstrating that methionine, via SLC43A2 and MATs, modulates histone methylation in immune cells, albeit with a distinct methylation pattern. Specifically, we found that methionine directly upregulates H3K4me3 at the *IL‐17A* and *IL‐22*, consistent with prior studies showing that elevated H3K4me3 suppresses *IL‐17A* secretion [27]. We also found that IL‐22 functions as a pro‐inflammatory mediator in ACRIII. Although previous studies have demonstrated the capacity of IL‐22 to enhance epithelial barrier integrity and restore tissue homeostasis, concurrent evidence from preclinical models indicates its paradoxical role in exacerbating inflammatory responses and potentiating oncogenic progression [28, 29]. Our findings corroborate the pro‐inflammatory paradigm of IL‐22 biology, particularly within the pathophysiological context of ACRIII. Our study revealed the role of methionine as a trigger for regulating this specific histone methylation process, providing novel insights into its immunomodulatory mechanisms. While DY801 may not be universally applicable, *metB*‐expressing bacteria could yield engineered probiotics with enhanced therapeutic potential.

However, this study had several limitations. First, whereas our mouse model offers insights into ACRIII mechanisms, whole‐abdomen irradiation does not fully replicate clinical colorectal targeting because current equipment requires a minimum field (~2 cm × 2 cm). This makes modeling localized colorectal injuries challenging. However, advancements in small‐animal radiotherapy technologies may enhance the clinical relevance of preclinical models. This study collected clinical samples from two centers in Guangdong, China, revealing a correlation between baseline *Lactobacillus* abundance and ACRIII incidence. However, given the known influence of geographical factors on gut microbiota composition, our findings may have limited generalizability. To address this, our team is conducting a multicenter clinical study that is expected to provide more robust and less biased evidence regarding the relationship between gut microbiota and ACRIII. Finally, functional validation of *Lactobacillus* strains derived from exceptional LARC patients, including *L. rhamnosus* DY801, has only been conducted at the preclinical level in mouse models. However, given that these strains are listed in the China National Catalog of Edible Microorganisms, they have the potential to be rapidly translated into clinical applications. To bridge this gap, our team initiated clinical studies to evaluate their roles in alleviating ACRIII, improving patient quality of life, and reducing the burden of cancer treatment, with a focus on efficacy, feasibility, cost‐benefit analysis, and patient‐reported outcomes.

## CONCLUSION

Our study identifies *Lactobacillus* strains isolated from LARC patients with CR and resistance to ACRIII as a novel class of microbial therapeutics. Functional characterization of ten patient‐derived strains, notably *Lactobacillus rhamnosus* DY801, reveals a microbial‐metabolite axis wherein methionine synthesis (*metB*‐dependent) epigenetically reprograms gut Lti cells through H3K4me3‐mediated chromatin remodeling, suppressing pro‐inflammatory cytokines IL‐17A and IL‐22 without compromising antitumor efficacy. This mechanism positions patient‐derived probiotics as precision biotherapeutics for ACRIII prevention.

By establishing a translational pipeline for probiotic discovery, we demonstrate how the microbiota from exceptional responders mitigates treatment toxicity. Future work will evaluate oral DY801 consortia in clinical trials for ACRIII prophylaxis, while mechanistic studies will delineate microbial‐epigenetic crosstalk in inflammatory comorbidities, informing strategies to improve therapeutic outcomes.

## METHODS

The reagents used in this study are listed in Table S4.

### In‐house patient and sample collection

The participants clinical information was reported by the physician, as shown in Table S5. Data on socioeconomic status, ethnicity, and race or ancestry were not collected. Patient specimens and data were collected according to the guidelines of the Ethics Committee of the Nanfang Hospital, Southern Medical University (NFEC‐2023‐219) and the Ethics Committee of General Hospital of Southern Theatre Command (NZLLKZ2025003). Informed consent was obtained, allowing patients to withdraw from the study. Eligible patients included those aged 18–75 years with mismatch repair proficiency or genetic testing indicating microsatellite stability of locally advanced rectal adenocarcinoma, as confirmed by pathological histology and imaging without randomization and blinding. The detailed inclusion and exclusion criteria are listed in Table S6. All enrolled patients received the same standardized neoadjuvant chemoradiotherapy: concurrent long‐course radiotherapy (50 Gy/25 fractions, 2 Gy/fraction, 5 days/week) and chemotherapy (capecitabine: 825 mg/m², bid, po, days 1–5 during radiotherapy; 1000 mg/m², bid, po, Days 1–14, q3w post‐radiotherapy; CapeOX: Oxaliplatin 130 mg/m² plus capecitabine 1000 mg/m², bid, Days 1–14, q3w). The clinical efficacy was evaluated preoperatively (at least 8–10 weeks post‐radiotherapy) via physical examination, colonoscopy, MRI (rectal), and CT (chest, abdomen, and pelvis). Patients achieving cCR after neoadjuvant therapy could choose the “watch‐and‐wait” strategy without surgery. Patients who did not achieve cCR proceeded to total mesorectal excision surgery if indicated or received further chemotherapy with re‐evaluation. The system used to grade ACRIII was the Common Terminology Criteria for Adverse Events (CTCAE) (Table S7). Patients showing no signs of ACRIII or graded as 1 were designated as non‐ACRIII, whereas all the remaining patients were classified as ACRIII. None of the patients received probiotics, antibiotics, prebiotics, immunosuppressants, or corticosteroids during the 4 weeks preceding the sample collection.

### In vivo mouse models

All animal experiments were approved by the Institutional Animal Care and Use Committee of the Nanfang Hospital, Southern Medical University. Six‐ to 7‐week‐old male C57BL/6J mice (18–20 g) were obtained from the Experimental Animal Center of the Southern Medical University and raised under specific pathogen‐free conditions with ad libitum access to food and water. Mice were allowed to acclimatize to the experimental environment for 1 week. They were randomly assigned to groups using simple randomization without blinding. The ACRIII mouse model was established using a total abdominal irradiation dose of 6 Gy (field: 3 × 20 cm, 512 cGy/min; Varian Clinac 23EX Linear Accelerator) and three intraperitoneal injections of 5‐fluorouracil (50 mg/kg/day). Fresh feces were collected before irradiation and stored at −80°C for FMT treatment. Fecal samples (200 mg) were resuspended in 1 mL of sterile anaerobic phosphate‐buffered saline (PBS) and centrifuged at 800×*g* for 5 min. The supernatant was passed through a 70‐μm filter to remove large particulates. Mice received a 0.2 mL fresh fecal solution intragastrically once daily for 3 d. For *Lactobacillus*, *Akkermansia muciniphila*, and DY801‐△metB treatments, mice were intragastrically administered 1 × 10⁹ CFU/200 µL/day for 7 days. For loperamide treatment, mice were intragastrically administered 5 mg/kg loperamide for 3 days. The mice were administered 200 mg/L of drinking water daily for ampicillin treatment. For the *Lactobacillus* combination treatments (CYQ09 + DY801, DY801 + DY802, CYQ09 + DY802, and 3Mix of DY801 + DY802 + CYQ09), mice were intragastrically administered a total *Lactobacillus* dose of 1 × 10⁹ CFU (in 1:1 or 1:1:1 ratios) suspended in 200 µL of sterile PBS daily for seven consecutive days. For the methionine‐deficient diet, mice were switched directly to the diet. GSK805 treatment involved oral administration of 30 mg/kg for 3 days. Mice receiving methionine treatment received 50 mM l‐methionine added to their drinking water. Mice treated with SAM or cLeu were administered with 5 mg/kg SAM or 2.5 mg/kg cLeu (dissolved in sterile PBS) daily. Body weight and survival were monitored throughout the experiment.

To establish the orthotopic tumor model, the mice were anesthetized and subjected to laparotomy on Day ‐7. In the cecum triangle region, 5 × 10⁵ MC38 cells transfected with mCherry fluorescent virus were inoculated. On Day ‐1, in vivo imaging assessed the peritoneal tumor size. Mice with unusual tumor sizes were excluded, and those with similar baseline sizes were selected for further experiments. On Day 0, the ACRIII model was constructed as previously described. From Day ‐7 onward, the mice received DY801 or ATCC 53103 at the same bacterial doses. The mice were inoculated with 1 × 10⁷ MC38 cells in the dorsal flank on Day ‐7 for the subcutaneous tumor model. From Day ‐7, DY801 or ATCC 53103 was administered orally daily. Body weight and survival were monitored throughout the study.

### Bacterial culture and cell culture

Totally, 10 novel *Lactobacillus* strains with stable genetic profiles were isolated from fresh fecal samples of seven exceptional patients who achieved CR without ACRIII. Fecal homogenates were plated on MRS agar and cultured under the strict anaerobic conditions (80% N_2_, 10% H_2_, and 10% CO_2_) in an anaerobic chamber (Don Whitley Scientific, West Yorkshire, UK) at 37°C for 24–48 h. Single colonies were purified and identified using 16S rRNA gene sequencing. All *Lactobacillus* strains in the other experiments were cultured under the same strictly anaerobic conditions (80% N_2_, 10% H_2_, and 10% CO_2_) at 37°C in MRS culture medium. *Akkermansia muciniphila* was cultured under the same conditions, except that BHI culture medium was supplemented with 0.2% mucin. MC38 cells were cultured in high‐glucose DMEM containing 10% FBS, 50 U/mL penicillin, and 50 mg/mL streptomycin, and maintained at 37°C in a 5% CO_2_ incubator.

### Primary intestinal epithelial cell isolation and co‐incubation

Pregnant C57BL/6 females on gestation Days 16–19 were euthanized by cervical dislocation. After disinfection with 75% ethanol, intrauterine fetal mice were dissected on a clean bench. Intestinal segments of the fetuses were cut into pieces of approximately 1 mm², resuspended in 1 mL of DMEM/F‐12 medium, and centrifuged at 250×*g* for 5 min. The supernatant was discarded, and the tissue was resuspended in 4 mL DMEM/F‐12 medium containing 5% FBS, 2 mM l‐glutamine, 10 ng/mL EGF, and 5 µg/mL insulin with antibiotics. The suspension was cultured in a 6‐well dish for 3–5 days. Once adhered, primary intestinal epithelial cells were obtained using a differential digestion method. Following co‐incubation with bacterial supernatants, cells were washed with PBS to remove the original medium and incubated with 500 μL of bacterial supernatant (1 × 10⁶ CFU/mL) for 6 h, after which the cells were collected for subsequent experiments.

### Metagenomic sequencing data analysis

Human and mouse feces were prospectively collected at baseline following the International Human Microbiome Standards. Fresh samples were stored in sterile 50 mL containers and frozen at −80°C until DNA extraction. A Stool DNA Isolation Kit (Magnetic Beads) was used for microbial genomic DNA extraction. DNA purity and concentration were assessed using NanoDrop 2000 (Thermo Fisher Scientific) and Quantus Fluorometer (Picogreen, Invitrogen Corporation), respectively. DNA integrity was evaluated using 1% agarose gel electrophoresis. One milligram of genomic DNA was used for the library construction. The DNA was ultrasonically sheared into 350 bp fragments using a focused ultrasonicator (ME220, Covaris). A paired‐end library was constructed using NEXTFLEX Rapid DNA‐Seq (Bioo Scientific). Paired‐end sequencing was conducted using an Illumina NovaSeq™ X Plus (Illumina Inc.) at Majorbio Bio‐Pharm Technology Co. Ltd., using the NovaSeq X Series 25B Reagent Kit according to the manufacturer's instructions.

The data processing and figure visualization were performed using the free online Majorbio Cloud Platform [30]. Raw sequencing reads were trimmed for adapters and low‐quality reads (length < 50 bp or average quality value < 20 bp) using fastp [31]. Reads were aligned to the human genome using BWA and hits associated with the reads and their mates were removed [32]. Quality‐filtered data were assembled using the MEGAHIT software [33]. Contigs ≥ 300 bp were selected as the final assembly results. Open reading frames (ORFs) from each assembled contig were predicted using Prodigal, and ORFs ≥ 100 bp were retrieved. A nonredundant gene catalog was constructed using CD‐HIT, with 90% sequence identity and coverage [34]. Gene abundance for each sample was estimated using the SOAPaligner at 95% identity [35]. The best‐hit taxonomy of nonredundant genes was obtained by aligning them against the NCBI NR database using DIAMOND with an e‐value cutoff of 1e^−5^ [36]. KEGG functional annotation of nonredundant genes was similarly performed. Alpha and beta diversity, principal coordinate analysis (PCoA), and species and KEGG functional contribution analyses between groups were conducted at the species or genus level. LEfSe was performed on the two groups at the genus level.

### Intestinal permeability assay

The mice were fasted for 4 h before measurement and then administered 50 mg/100 g of 4KD FITC‐dextran. Two hours later, 0.2 mL of blood was collected using the inner‐canthus bleeding method. Blood samples were centrifuged at 3000×*g* for 15 min to collect serum, and fluorescence was analyzed with a Synergy Neo2 Multi‐Mode Microplate Reader (BioTek Instruments). All procedures were performed in the dark to prevent fluorescence quenching, with excitation and emission wavelengths of 490 and 520 nm, respectively. The serum from normal mice was used as the reference standard.

### HE staining and histopathological analysis

Mouse intestinal tissues were fixed overnight in 4% paraformaldehyde. Tissues were dehydrated using an automated system and embedded in paraffin. Four‐micrometer sections were prepared for HE staining. The slides were baked at 65°C for 4 h and deparaffinized in xylene (15 min each, three times). The tissues were rehydrated using ethanol solutions (absolute 95%, 90%, 80%, and 70%) for 5 min each. Tissues were washed with PBS, stained with hematoxylin for 5 min, differentiated in 1% acidic ethanol for 5 s, rinsed with water, and counterstained with eosin for 3 min. The samples were dehydrated in an ethanol series (70%, 80%, 90%, and 95% absolute for 30 s) and cleared twice with xylene (2 min each). The tissues were mounted with neutral gum and examined under a microscope. Villus length was measured from the villus tip to the crypt opening, and from the crypt opening to the bottom. At least 50 villi across five fields of view using ImageJ software.

### Immunofluorescence staining

Primary intestinal epithelial cells were seeded in six‐well plates. The cells were then washed with PBS and fixed in 4% paraformaldehyde overnight. After discarding the fixation solution, the fixed cells were washed three times with PBS for 3 min each on a shaker. The cells were permeabilized with 0.5% Triton X‐100 for 15 min, followed by three PBS washes for 3 min each. The PBS was removed, and the cells were blocked with 5% BSA for 30 min at room temperature. The cells were then incubated with a diluted anti‐CK‐18 antibody (1:300) at 4°C overnight. The primary antibody was discarded, and the cells were washed three times with PBS containing 0.1% Tween‐20 (PBST) for 3 min each. Subsequently, diluted goat Anti‐Rabbit IgG H&L (Alexa Fluor® 594, 1:1000) was added and the cells were incubated for 30 min in a dark, moist chamber. The cells were washed three times with PBST for 3 min each time. DAPI staining solution was added, and the cells were examined under a fluorescence microscope (BX51; Olympus Corporation).

### *Lactobacillus* screening experiment

*Lactobacillus* screening experiments were performed as previously described with minor modifications [37].

#### Acid and bile salt tolerance

Artificial gastric juice was prepared using hydrochloric acid and pepsin at pH 2.0, 3.0, and 4.0, and artificial intestinal fluid containing bile salts was prepared using trypsin and bile salts at pH 6.8. Bacteria were diluted in sterile PBS to a concentration of 10^9^ CFU/mL. A 1 µL aliquot of the bacterial suspension was added to 99 µL of artificial gastric juice at different pH values and cultured anaerobically in a 96‐well plate at 37°C for 3 h. The cultured suspension was plated onto MRS agar and incubated for 48 h to count CFUs.

##### Cell adhesion assay

Primary intestinal epithelial cells were digested and seeded in a six‐well plate to form a monolayer. Bacteria were centrifuged at 8000×*g* for 5 min to obtain a pellet, which was resuspended in sterile PBS and diluted to an OD_600_ of 1. After removing the culture medium, the bacterial suspension (0.5 mL) was added and the coculture was performed at 37°C for 6 h. After incubation, cells were washed three times with PBS, scraped off, and diluted ten‐fold for plating on MRS agar, incubated at 37°C for 48 h, and the adhesion rate was calculated based on the CFU.

##### Cell viability assay

Bacteria were collected and resuspended in 1 g/mL sterile PBS and heated (100°C for 20 min) for inactivation. Primary intestinal epithelial and MC38 cells were digested, counted, and seeded in a 96‐well plate at 2000 cells per well. Following attachment, the culture medium was replaced with 0.1 mL of inactivated bacteria or the bacterial supernatant. After 48 h of incubation, the supernatant was discarded, and 0.1 mL of CCK‐8 solution diluted in DMEM/F‐12 or DMEM was added. After 4 h of incubation, OD_450_ was measured to determine cell viability.

##### Comet assay

Primary intestinal epithelial cells were seeded in a six‐well plate and allowed to adhere before replacing the medium with 1 mL of bacterial supernatant (maintaining a consistent 1 × 10^6^ CFU/mL). The cells were then subjected to a single 6 Gy irradiation dose. After 4 h, the cells were digested, washed once with PBS, and resuspended at a density of 1 × 10^6^ cells/mL. The comet assay was performed using a comet assay kit according to the manufacturer's instructions, starting with the first layer of 1.5% normal melting point agarose on a slide, followed by the second layer of 0.7% low‐melting‐point agarose containing 10 µL of cells, and topped with the third layer of 1.5% normal melting point agarose. The slides were immersed in pre‐chilled lysis buffer at 4°C for 2 h, washed with PBS for 5 min, and placed in an electrophoresis chamber filled with 1 L of TAE buffer for 30 min. After DNA unwinding, slides were subjected to electrophoresis at 100 V for 15 min in the dark. After electrophoresis, 10 µL of propidium iodide (PI) staining solution was added to the agarose gel. Coverslips were applied, and the slides were observed and photographed under a fluorescence microscope to ensure that 50 comets were randomly selected. The percentage of comet tail DNA was analyzed using CASP 1.2.3.

##### Colony assay and multitarget single‐hit model

MC38 cells were digested to obtain a cell suspension, which was diluted in a six‐well plate at 400, 800, 1200, and 1600 cells/well. The following day, after cell adherence, the medium was replaced with 1 mL of fresh bacterial supernatant (1 × 10^6^ CFUs). The cells were irradiated with doses of 2, 4, 6, 8, and 10 Gy (three replicates per dose). Four hours, post‐irradiation, the medium was replaced with fresh complete medium, and the plates were incubated for 7 days. After washing with PBS, cells were fixed in methanol for 30 min and stained with 0.1% crystal violet for 15 min. Using a double‐blind method, the number of colonies was counted, and the averages were calculated for each well. Relevant parameters were computed based on the multitarget single‐hit model (SF = 1 − (1 − e^−^^d/d0^)^N^), and the survival fraction curves were plotted.

##### IC_50_ determination

Primary intestinal epithelial cells and MC38 cells were digested, counted, and seeded in a 96‐well plate at 8000 cells/well. A dilution series of 5‐FU working solutions was prepared and added to cells. After 48 h, the supernatant was discarded, and 0.1 mL of CCK‐8 solution diluted in DMEM/F‐12 or DMEM was added. After 4 h of incubation, the OD_450_ was measured to calculate the IC_50_ of 5‐FU.

### Safety experiment

For acute toxicity testing, groups of mice received a single oral gavage of fresh *Lactobacillus* at a concentration of 1 × 10^10^ CFU/mL on day one. Mice were monitored for 14 days to assess clinical signs (behavior, appearance, diet, water intake, response to stimuli, secretions, and excretions), mortality (death, time of death, and pre‐death reactions), and body weight changes. On Day 14, the mice were euthanized, and vital organs and fresh blood were collected for analysis. Blood samples were divided as follows: one mixed with EDTA anticoagulant for blood cell counts using a hematology analyzer (BC‐5000VET, Mindray) and the other centrifuged at 3000×*g* for 20 min for liver and kidney function analysis using a biochemistry analyzer (BS‐240 Vet, Mindray).

Antimicrobial susceptibility was determined using minimum inhibitory concentration (MIC) assays following the European Committee on Antimicrobial Susceptibility Testing (EUCAST) guidelines. Antibiotics were prepared at concentrations as outlined in the 2022 EUCAST guidelines: tetracycline (0.25–128 μg/mL), streptomycin (2–1024 μg/mL), gentamicin (2–1024 μg/mL), vancomycin (1–512 μg/mL), cefoperazone (1–512 μg/mL), amoxicillin (0.0625–32 μg/mL), meropenem (0.0625–32 μg/mL), ciprofloxacin (1–512 μg/mL), and metronidazole (2–1024 μg/mL). Sulfasalazine and loperamide were prepared at 2–1024 μg/mL using two‐fold dilutions. Bacterial suspensions (1 × 10^6^ CFU) were inoculated into 96‐well plates containing various antibiotic concentrations and incubated at 37°C under anaerobic conditions for 48 h. MICs were determined by measuring OD_600_ to identify the lowest concentration that inhibited visible growth.

### RNA extraction and quantitative PCR

Samples of 100 mg jejunal contents, bacteria, or jejunum were homogenized in 1 mL TRIzol reagent. RNA was extracted using the chloroform‐isopropanol method. RNA from the contents, bacteria, or tissues was reverse‐transcribed into cDNA using HiScript Q RT SuperMix for qPCR (+gDNA wiper) following the manufacturer's instructions. Subsequently, mRNA expression was analyzed using Taq Pro Universal SYBR qPCR Master Mix with a LightCycler 96 detection system (Roche). The 16S ribosomal RNA or GAPDH was used for normalization. The primers used for qPCR are listed in Table S4.

### Enzyme‐linked immunosorbent assay (ELISA)

The collected intestinal tissues were divided into jejunum, ileum, and colon segments. A 0.1 g portion of each tissue was added to 1 mL of PBS with 2 mm grinding beads for homogenization. The suspension was centrifuged at 12,000×*g* for 20 min at 4°C, and the supernatant was collected. Blood samples treated with EDTA were centrifuged at 3000×*g* for 20 min to obtain serum. Tissue homogenates and serum were analyzed for IL‐1β, IL‐6, IL‐10, TNF‐α, TGF‐β, IL‐17, and IL‐22 using ELISA kits. Briefly, 50 μL of homogenate, serum, or standards was added to each well and the reactions were conducted at 37°C for 30 min. Plates were washed five times, the enzyme conjugate was added, and the reaction was continued for another 30 min at 37°C. After washing, chromogenic reagents A and B were added and the reaction was allowed to proceed for 10 min at 37°C. A stop solution was added, and the optical density at OD_450_ was measured using a microplate reader. A standard curve was generated to calculate the cytokine levels in the samples.

### In vivo imaging technique

Mice were fasted and deprived of water for 24 h before the experiment, and depilatory cream was applied to minimize interference with the fluorescent signals. On the experimental day, the mice were anesthetized with isoflurane and placed in the imaging chamber of an in vivo imaging system (FX Pro; Bruker). Fluorescence was excited using a far‐red light source at 760 nm, and images were captured for fluorescent area quantification using supporting software.

### Bacterial whole genome sequencing and comparative genomics analysis

Single colonies of DY801 were isolated via streaking and culturing. A 5 g bacterial pellet was collected for genomic DNA extraction using standard protocols from Oxford Nanopore Technologies. DNA purity, concentration, and integrity were assessed using NanoDrop, Qubit, and 0.35% agarose gel electrophoresis. Large DNA fragments were recovered using a BluePippin automated DNA recovery system. Library preparation was performed using a SQK‐LSK109‐XL Ligation Sequencing Kit. Library quantification was performed using the Qubit software. Sequencing was performed on a Nanopore platform, and the data were converted to the FASTQ format using Guppy v3.2.6. Low‐quality and short reads (<2000 bp) were filtered out. Filtered reads were assembled using Canu v1.5 [38], corrected with Racon v3.4.3 [39], and circularized or adjusted using Circlator v1.5.5 [40]. Error correction was performed using Pilon v1.2246 with [41] 16S rDNA gene sequencing data to obtain the complete genome sequence. The DY801 genome was compared with the publicly available ATCC53103 genome. Gene prediction was performed using Prodigal v2.6.3 [42], followed by homologous and unique gene analysis. Differential gene analysis and KEGG pathway annotation were performed using the ClusterProfiler R package [43]. Biomarker Technologies Co. Ltd. conducted all the data processing and figure visualization.

### Untargeted metabolomics

Untargeted metabolomic analysis of the mouse fecal samples was conducted by Majorbio Bio‐Pharm Technology Co. Ltd. Fecal samples (100 mg per group) were homogenized in 100 μL of sterile PBS. The homogenate was centrifuged at 12,000×*g* for 10 min and the supernatant was collected. Metabolites were extracted using methanol and acetonitrile, filtered through a 0.22 μm filter, and subjected to LC‐MS analysis. Raw data were processed for missing‐value imputation and data normalization. Identified metabolites were annotated by comparison against the KEGG and HMDB databases, with statistical analysis performed on the annotated metabolites. The data processing and figure visualization were performed using the free online Majorbio Cloud Platform [30].

### Bacterial gene knockout

#### Vector construction

sgRNA targeting the *metB* gene within the *cysE*‐*metB*‐*cysM* cluster of DY801 was designed using the CHOPCHOP website. Primers and Phanta Max Super‐Fidelity DNA Polymerase amplified approximately 1 kb upstream and downstream homologous arms of DY801 DNA. sgRNA and scaffolds were amplified from the pLCNICK plasmid by replacing the original sgRNA sequence. The linear plasmid was cloned with the sgRNA scaffold and homologous arms using the ClonExpress® MultiS One Step Cloning Kit, incubated at 37°C for 30 min, and then stopped at 4°C. A 10 μL aliquot was transformed into *E. coli* DH10B cells, plated on LB agar with 50 μg/mL kanamycin, and incubated at 37°C until a single colony appeared. Positive clones were screened by colony PCR, and recombinant plasmids were extracted and stored at −20°C.

##### Preparation of competent cells

*Lactobacillus rhamnosus* DY801 was resuscitated and passed once before inoculation into 50 mL of MRS liquid medium. After culturing at 37°C for 12 h, a 10 mL aliquot was transferred into 100 mL of MRS containing 2.5% glycine. Cultivation ceased when the OD_600_ reached 0.5–0.6, and the cells were harvested by centrifugation at 8000×*g* for 5 min at 4°C. The cell pellet was washed twice with pre‐chilled sterile water, resuspended in pre‐chilled 0.05 mM EDTA solution, incubated on ice for 5 min, and collected by centrifugation. The cells were washed twice with pre‐chilled sterile water and resuspended in pre‐chilled electroporation buffer. After a final wash with electroporation buffer, the cells were resuspended in 0.8 mL electroporation buffer and aliquoted into 0.1 mL portions, completing the preparation of competent DY801 cells.

##### Electroporation of plasmids

The knockout plasmid (10 μL) was gently mixed with competent cells and incubated on ice for 5 min before being transferred to a pre‐chilled 0.1 mm electroporation cuvette. Electroporation was conducted at 1.25 kV with a single pulse using the Gene Pulser Xcell Electroporation System (Bio‐Rad). Immediately after electroporation, 890 μL of prewarmed MRS medium was added, mixed gently, and incubated at 37°C for 4 h for bacterial recovery. Cells were collected by centrifugation at 8000×*g* for 5 min, resuspended in 100 μL of MRS medium, and plated on MRS agar containing 500 μg/mL kanamycin.

##### Screening and identification

Single colonies from the plates were inoculated into liquid MRS containing 500 μg/mL kanamycin and incubated overnight. Half of the culture was centrifuged, and genomic DNA was extracted using the Bacterial DNA Isolation Mini Kit. PCR was performed using primers flanking the upstream and downstream homologous arms, genomic DNA, and 2 × Taq Master Mix. For *metB*‐knockout strains, the expected amplicon size was approximately 2 kb, whereas non‐knockout strains yielded a product around 3.2 kb, corresponding to a *metB* gene size of 1184 bp. Amplified fragments from putative *metB*‐knockout strains were sent for sequencing verification. The successfully generated *metB‐knockout* strains were designated as DY801‐ΔmetB.

### Liquid chromatography‐mass spectrometry (LC‐MS)

Accurately weighing 2.5 mg of methionine, homocysteine, SAM, and SAH (HPLC grade) dissolved in 1 mL of Milli‐Q water with 0.1% formic acid yielded a stock solution of 2.5 mg/mL. Dilution with acetonitrile (v/v, 5/95) prepared standard solutions of methionine and homocysteine at 0.5, 2, 5, 10, 25, 50, 100, and 500 μM and SAM and SAH at 0.125, 0.5, 2, 5, 10, 25, 50, 100, and 500 nM. A standard curve was generated based on the peak areas.

Homogenization of 0.1 g of intestinal tissue, bacteria, or feces with 0.1 mL Milli‐Q water and 2 mm grinding beads was followed by centrifugation at 12,000 ×*g* to collect the supernatant in clean tubes. For serum analysis, 50 μL of sample was mixed with 150 μL of 1% formic acid in methanol and vortexed for 5 min. After centrifugation at 14,000 ×*g* at 4°C for 5 min, the supernatant was filtered through a 0.22 μm filter and transferred to a mass spectrometry vial.

A UPLC XSelect HSST3 column was used at a flow rate of 0.4 mL/min with a 10 μL injection volume and a column temperature of 30°C in a Thermo TSQ Quantum Ultra Triple Quadrupole LC‐MS/MS (Thermo Fisher Scientific). Mobile phase A was 0.1% formic acid in water, and phase B was acetonitrile. The elution program lasted for 13 min. Concentrations were calculated from the sample peak areas using a standard curve.

### Pan‐genomics analysis

The reference genomes of 1520 cultivated human gut bacteria were sourced from the NCBI database (No. PRJNA48274833) [44]. De novo assembly was performed as previously described [44]. Gene and protein predictions in these bacterial genomes used Prokka (v1.3) [45] to extract taxonomic information from strain names. The *metB* gene was selected for further analyses.

### Bulk RNA sequencing

Total RNA was extracted from the jejunum using TRIzol reagent. Approximately 60 mg of the intestine was powdered in liquid nitrogen, homogenized, and centrifuged for RNA isolation. Chloroform/isoamyl alcohol was used for RNA extraction, followed by precipitation with isopropyl alcohol. Purified RNA was quantified and its quality was assessed using NanoDrop and Agilent 2100 (Thermo Fisher). mRNA was isolated using oligo(dT)‐attached beads, fragmented, and reverse‐transcribed into cDNA. The cDNA was purified, end‐repaired, and ligated with adapters for PCR amplification. The quality and quantity of the cDNA library were checked using Agilent 2100 and qPCR. The library was subjected to duplex‐specific nuclease treatment, flow‐cell amplification, and single‐end sequencing on an Illumina NovaSeq. 6000 platform to obtain high‐quality sequencing data. Genes with a |log_2_ (fold change)| ≥ 1 and a *q* value < 0.05 were designated as DEGs. KEGG pathway enrichment analysis of these genes was performed using the ClusterProfiler R package. The data processing and figure visualization were performed using the free online Majorbio Cloud Platform [30].

### Spatial transcriptomics and gene set variation analysis (GSVA)

Spatial transcriptomics primarily uses a Digital Spatial Profiling Instrument (NanoString Technologies) by Fynn Biotechnologies (Shandong) Co., Ltd. with an accompanying PCR amplification reagent kit for library construction and product purification. Intestinal tissues from the Control, RCT, and DY801 groups were prepared as 4 µm‐thick paraffin sections, deparaffinized, and hydrated according to the DSP‐standard protocol. Antigen retrieval was performed using Tris‐EDTA buffer in a pressure cooker, followed by proteinase K digestion. The sections were fixed with formaldehyde at room temperature for 5 min and incubated overnight in the dark with anti‐CD45 rabbit mAb and anti‐RORγ rabbit mAb in a hybridization oven. After blocking in the dark, nuclear staining was performed using the DSP‐standard Nuclear Stain SYTO13, followed by instrument detection. All sections were scanned using DSP, and ROIs were selected from within the lamina propria of the small intestine. After ROI selection, DSP technology decomposed the oligos through UV irradiation. The decomposed oligos were collected and concentrated on a collection plate sealed with a breathable membrane. The collection plate was dried in a PCR machine, resuspended in DPEC water, briefly centrifuged, and subjected to PCR using the DSP‐standard protocol for library construction. The library was purified using magnetic beads to obtain a clean, sequencing‐ready library. The purified product was quantified using QFX, and the fragment length was assessed using Qsep100. The library was sequenced according to the standard DSP protocol requirements. For sequencing data from each ROI, abundance analysis of cell subtypes was performed using single‐cell sequencing data (GSE 124880) [46] and GSVA R package [47]. Genes with a |log_2_ (fold change)| ≥ 1 and a *q* value < 0.05 were designated as DEGs. KEGG pathway enrichment analysis of these genes was performed using the ClusterProfiler R package [43]. The data processing and figure visualization were performed by Fynn Biotechnologies (Shandong) Co., Ltd.

### Flow cytometry sorting

To sort the Lti cells, the small intestine of the mice was digested to obtain a single‐cell suspension. The cells were stained before sorting and purification using a FACSAria III Cell Sorter (BD Biosciences). The Lti cell gating strategy used was the live lineage^−^CD127^+^KLRG1^−^c‐kit^+^CCR6^+^. Sorted Lti cells were cultured in complete RPMI‐1640 medium supplemented with 10 ng/mL of recombinant human IL‐7, 10 ng/mL of recombinant human IL‐2, and 10 ng/mL of recombinant human SCF [48].

### Western blot

Nuclear and cytoplasmic proteins were extracted from 1 g of small intestinal tissue using the NE‐PER™ Nuclear and Cytoplasmic Extraction Reagent kit. The protein concentration was determined using a NanoDrop spectrophotometer. Proteins were adjusted to a concentration of 10 mg/mL, mixed with 5× protein loading buffer, boiled for 10 min, and stored at −20°C. A 10% SDS‐PAGE gel was prepared by loading 30 mg of protein with a PageRuler™ Prestained Protein Ladder at the well ends. Electrophoresis was performed using a vertical system (Bio‐Rad Laboratories) at a constant voltage of 65 V for 15 min for the stacking gel and 95 V for 70 min for the resolving gel. For western blot analysis, the sandwich method was used for wet transfer at a constant current of 150 mA for 60 min. Membranes were blocked with 5% BSA at room temperature for 1 h. The PVDF membranes were incubated overnight at 4°C with primary antibodies specific for histone modifications (nuclear proteins) and methionine metabolism‐related genes (cytoplasmic proteins). The next day, the membrane was washed three times with PBST for 15 min each, followed by incubation with the corresponding secondary antibodies (diluted 1:2000 in PBST) for 2 h. The membrane was washed three times with PBST for 15 min each. ECL detection was performed using Tanon™ Femto‐sig ECL Western blot analysis. Substrate and images were captured using a Tanon 5200 Series Fully Automatic Chemiluminescence Image Analysis System (Tanon).

### Cellular gene knockdown

The Lti cells were collected and adjusted to a concentration of 3 × 10^6^ cells/mL, followed by two washes with PBS. Cells were resuspended in Opti‐MEM and mixed with 4 µg plasmid DNA. The mixture was transferred to a 0.4 cm electroporation cuvette and subjected to electroporation (350 V, 975 μF, 1 pulse, Gene Pulser Xcell Electroporation Systems; Bio‐Rad). After electroporation, cells were transferred to a 24‐well plate and cultured in RPMI‐1640 medium supplemented with 10% FBS. After 48 h, 4 µg/mL of puromycin was added for selection. After 48 h, the cells were collected for subsequent experiments.

### CUT&Tag

The CUT&Tag assay was performed using the Hyperactive Universal CUT&Tag Assay Kit following the manufacturer's instructions. Lti cells were collected and adjusted to a density of 10,000 cells/tube. Cell pellets were resuspended in 100 µL of Wash Buffer and transferred to an 8‐Strip PCR tube containing activated ConA Beads Pro. The mixture was incubated for 10 min with gentle mixing. Next, 50 µL of cold Antibody Buffer was added to resuspend the cell (nuclei)‐bead complexes, and H3K4me3 antibody was added at 1:100 dilution for overnight incubation at 4°C. The next day, 50 µL of secondary antibody diluted in Dig‐Wash Buffer was added. The beads were washed twice, and 100 µL of pre‐diluted pA/G‐Tnp Pro was added for 1‐h incubation at room temperature, followed by two additional bead washes. Next, 50 µL of diluted TTBL was added, and the samples were incubated at 37°C for 60 min for fragmentation. The reaction was stopped by adding 2 µL of 10% SDS and an appropriate amount of DNA Spike‐in, followed by incubation at 55°C for 10 min. The supernatant was collected, and DNA was extracted using DNA Extract Beads Pro. The resulting DNA was amplified into an Illumina library and purified using VAHTS DNA Clean Beads. The PCR products were subjected to Illumina sequencing for further analysis. CUT&Tag sequencing and figure visualization were performed by Sangon Biotechnology Co. Ltd.

### 16S rRNA gene sequencing

Fecal samples from patients in cohort 3 with LARC were stored at −80°C until processing. RNA was extracted using a Fecal RNA Extraction Kit, following the manufacturer's protocol. Briefly, 0.25 g of fecal material was homogenized in lysis buffer, and RNA was isolated via phenol‐chloroform extraction and silica column purification. The sequencing data quality was assessed using Trimmomatic (v0.33) [49] to remove low‐quality reads and adapters. Primer sequences were trimmed using Cutadapt (v1.9.1) with a maximum mismatch rate of 20% and a minimum overlap of 80%. Paired‐end reads were assembled using USEARCH (v10) [50] with a minimum overlap of 10 bp and 90% similarity. Chimeric sequences were removed using UCHIME (v8.1) [51]. High‐quality sequences were clustered into OTUs at 97% similarity using USEARCH and sequences representing <0.005% of the total reads were discarded. For a higher resolution, denoising and ASV generation were performed using DADA2 in QIIME2 (v2020.6) [52] with a 0.005% read filter. Taxonomic classification of OTUs and ASVs was conducted using the SILVA database (v138) [53] with the classification‐sklearn algorithm in QIIME2. Poorly aligned sequences were classified using classification consensus blast with 90% similarity and coverage. Alpha diversity indices (e.g., ace index) and beta diversity analyses (e.g., PCA based on Bray–Curtis and unweighted UniFrac distances) were performed in QIIME2 and R. The data processing and figure visualization were performed using the free online Majorbio Cloud Platform [30].

### Statistical analysis

Data are presented as means ± SEM. Statistical analyses were performed using Prism 8.0 (GraphPad version 8). Specifically, two‐tailed unpaired *t*‐test and Wilcoxon rank‐sum test between two groups, one‐way analysis of variance (ANOVA) and two‐way ANOVA across multiple groups were used to determine significance. For time‐course analysis of body weight, two‐way ANOVA was used. Survival was analyzed with the Mantel–Cox log‐rank test. Statistical parameters were represented in the Figure Legend of each Figure. *p* < 0.05 was considered significant. *p < 0.05, **p < 0.01, and ***p < 0.001.

## AUTHOR CONTRIBUTIONS

**Lu Yu**: Data curation; writing—original draft; investigation; validation; writing—review and editing. **Zhenhui Chen**: Data curation; funding acquisition; writing—original draft; investigation; software; writing—review and editing. **Shengqi Yin**: Validation. **Qiqing Guo**: Data curation. **Yuchuan Chen**: Data curation. **Jiaying Li**: Data curation. **Yafang Wang**: Formal analysis. **Xiangqiang Liu**: Resources. **Zi Xu**: Formal analysis. **Yaowei Zhang**: Funding acquisition; Visualization. **Yuqin Zhang**: Funding acquisition; Visualization. **Zhihao Zheng**: Visualization. **Keli Chen**: Visualization. **Yanqing Ding**: Supervision. **Hongying Fan**: Supervision; writing—review and editing. **Zhifeng Liu**: Supervision; writing—review and editing. **Yi Ding**: Supervision; funding acquisition; writing—review and editing.

## CONFLICT OF INTEREST STATEMENT

The authors declare no conflicts of interest.

## ETHICS STATEMENT

Participants' socioeconomic status, ethnicity, and race/ancestry were not collected. Patient specimens and data were gathered per the guidelines of the Ethics Committee of Nanfang Hospital, Southern Medical University (NFEC‐2023‐219) and the Ethics Committee of General Hospital of Southern Theatre Command (NZLLKZ2025003). All animal experiments were approved by the Institutional Animal Care and Use Committee of Nanfang Hospital, Southern Medical University (NFYY‐2018‐0521). This study meets our standards of animal ethics.

## AI STATEMENT

Deepseek‐V3 has been used to polish the language of the manuscript.

## Supporting information


**Figure S1.** Gut microbiota influences acute chemoradiotherapy‐induced intestinal injury (ACRIII).
**Figure S2.** Screening of *Lactobacillus* strains derived from exceptional rectal cancer patients.
**Figure S3.** Safety of *Lacticaseibacillus rhamnosus* (*L. rhamnosus*) DY801.
**Figure S4.** Exploration of the single and combined effect of novel strains from exceptional patients in alleviating ACRIII.
**Figure S5.** *L. rhamnosus* DY801 inhibits inflammation and enhances tumor therapy for subcutaneous tumors.
**Figure S6.** Pan‐genomic analysis and function of the *metB* gene.
**Figure S7.** *In vitro* and in vivo experiments with lymphoid tissue inducer (Lti) cells.
**Figure S8.** Cut & Tag sequencing of Lti cells treated with methionine or knockdown of genes.
**Figure S9.** *In vivo* experiments validate that S‐adenosylmethionine (SAM) affects the length of jejunal villi and cyrpt.

**Table S1.** The enriched pathways of human feces metagenomics.
**Table S2.** The area under the curve (AUC) of different number of variables using Random Forest.
**Table S3.** The enriched pathways of mice jejunum bulk RNA‐seq.
**Table S4.** Key resources table.
**Table S5.** Baseline characteristics of patients with rectal cancer enrolled in this study.
**Table S6.** Additional detailed inclusion and exclusion criteria.
**Table S7.** Grading of acute chemoradiotherapy‐induced intestinal injury (ACRIII).

## Data Availability

The metabolomics data reported in this paper have been deposited in the OMIX, China National Center for Bioinformation (CNCB) under accession number OMIX009383 (https://ngdc.cncb.ac.cn/omix/release/OMIX009383). All the sequencing data supporting the findings of this study are available in the China National Center for Bioinformation (CNCB) under accession numbers PRJCA031547 (https://ngdc.cncb.ac.cn/bioproject/browse/PRJCA031547) and CRA022263 (https://ngdc.cncb.ac.cn/gsa/browse/CRA022263). The data and scripts for analysis and visualization are saved in GitHub https://github.com/yulugithub/iMeta. Supplementary materials (figures, tables, graphical abstract, slides, videos, Chinese translated version, and update materials) may be found in the online DOI or iMeta Science http://www.imeta.science/.

## References

[1] Fernandez, Laura M. , Guilherme P. São Julião , Nuno L. Figueiredo , Geerard L. Beets , Maxime J. M. van der Valk , Renu R. Bahadoer , Denise E. Hilling , et al. 2021. “Conditional Recurrence‐Free Survival of Clinical Complete Responders Managed by Watch and Wait After Neoadjuvant Chemoradiotherapy for Rectal Cancer in the International Watch & Wait Database: A Retrospective, International, Multicentre Registry Study.” The Lancet Oncology 22: 43–50. 10.1016/s1470-2045(20)30557-x 33316218

[2] Serra‐Aracil, X. , C. Pericay , J. Badia‐Closa , T. Golda , S. Biondo , P. Hernández , E. Targarona , et al. 2023. “Short‐Term Outcomes of Chemoradiotherapy and Local Excision Versus Total Mesorectal Excision in T2‐T3ab, N0, M0 Rectal Cancer: A Multicentre Randomised, Controlled, Phase III Trial (The TAU‐TEM Study).” Annals of Oncology 34: 78–90. 10.1016/j.annonc.2022.09.160 36220461

[3] Bahadoer, Renu R. , Esmée A Dijkstra , Boudewijn van Etten , Corrie A. M. Marijnen , Hein Putter , Elma Meershoek‐Klein Kranenbarg , Annet G. H. Roodvoets , et al. 2021. “Short‐Course Radiotherapy Followed by Chemotherapy Before Total Mesorectal Excision (TME) Versus Preoperative Chemoradiotherapy, TME, and Optional Adjuvant Chemotherapy in Locally Advanced Rectal Cancer (RAPIDO): A Randomised, Open‐Label, Phase 3 Trial.” The Lancet Oncology 22: 29–42. 10.1016/s1470-2045(20)30555-6 33301740

[4] Fokas, Emmanouil , Anke Schlenska‐Lange , Bülent Polat , Gunther Klautke , Gerhard G. Grabenbauer , Rainer Fietkau , Thomas Kuhnt , et al. 2022. “Chemoradiotherapy Plus Induction or Consolidation Chemotherapy as Total Neoadjuvant Therapy for Patients With Locally Advanced Rectal Cancer: Long‐Term Results of the CAO/ARO/AIO‐12 Randomized Clinical Trial.” JAMA Oncology 8: e215445. 10.1001/jamaoncol.2021.5445 34792531 PMC8603234

[5] Conroy, Thierry , Jean‐François Bosset , Pierre‐Luc Etienne , Emmanuel Rio , Éric François , Nathalie Mesgouez‐Nebout , Véronique Vendrely , et al. 2021. “Neoadjuvant Chemotherapy With FOLFIRINOX and Preoperative Chemoradiotherapy for Patients With Locally Advanced Rectal Cancer (UNICANCER‐PRODIGE 23): A Multicentre, Randomised, Open‐Label, Phase 3 Trial.” The Lancet Oncology 22: 702–715. 10.1016/s1470-2045(21)00079-6 33862000

[6] Wang, Hongmiao , Huixun Jia , Yang Gao , Haosong Zhang , Jin Fan , Lijie Zhang , Fandong Ren , et al. 2022. “Serum Metabolic Traits Reveal Therapeutic Toxicities and Responses of Neoadjuvant Chemoradiotherapy in Patients With Rectal Cancer.” Nature Communications 13: 7802. 10.1038/s41467-022-35511-y PMC9759530 36528604

[7] Cristina, Valerie , Jestinah Mahachie , Murielle Mauer , Thierry Buclin , Eric Van Cutsem , Arnaud Roth , and Anna D. Wagner . 2018. “Association of Patient Sex With Chemotherapy‐Related Toxic Effects: A Retrospective Analysis of the PETACC‐3 Trial Conducted by the EORTC Gastrointestinal Group.” JAMA Oncology 4: 1003–1006. 10.1001/jamaoncol.2018.1080 29800044 PMC6145725

[8] Peng, Yu , Man Zhang , Lingjun Zheng , Qian Liang , Hanzeng Li , Jeng‐Ting Chen , Hongyan Guo , et al. 2017. “Cysteine Protease Cathepsin B Mediates Radiation‐Induced Bystander Effects.” Nature 547: 458–462. 10.1038/nature23284 28723894 PMC5892829

[9] Zhang, Cuiyu , Yuping Zhou , Junjie Zheng , Nannan Ning , Haining Liu , Wenyang Jiang , Xin Yu , et al. 2022. “Inhibition of GABAA Receptors in Intestinal Stem Cells Prevents Chemoradiotherapy‐Induced Intestinal Toxicity.” The Journal of Experimental Medicine 219: e20220541. 10.1084/jem.20220541 36125780 PMC9499828

[10] Shi, Xiaolin , Yiwei Yang , Wei Zhang , Jianxin Wang , Dexin Xiao , Huangge Ren , Tingting Wang , et al. 2022. “FLASH X‐Ray Spares Intestinal Crypts From Pyroptosis Initiated by cGAS‐STING Activation Upon Radioimmunotherapy.” Proceedings of the National Academy of Sciences of the United States of America 119: e2208506119. 10.1073/pnas.2208506119 36256824 PMC9618056

[11] Moraitis, Ilias , Jordi Guiu , and Josep Rubert . 2023. “Gut Microbiota Controlling Radiation‐Induced Enteritis and Intestinal Regeneration.” Trends in Endocrinology & Metabolism 34: 489–501. 10.1016/j.tem.2023.05.006 37336645

[12] Lam, Suk Yee , Maikel P. Peppelenbosch , and Gwenny M. Fuhler . 2019. “Prediction and Treatment of Radiation Enteropathy: Can Intestinal Bugs Lead the Way?” Clinical Cancer Research 25: 6280–6282. 10.1158/1078-0432.ccr-19-2422 31492747

[13] Cui, Ming , Huiwen Xiao , Yuan Li , Lixin Zhou , Shuyi Zhao , Dan Luo , Qisheng Zheng , et al. 2017. “Faecal Microbiota Transplantation Protects Against Radiation‐Induced Toxicity.” EMBO Molecular Medicine 9: 448–461. 10.15252/emmm.201606932 28242755 PMC5376756

[14] Lin, Shuang , and Yufei Shen . 2020. “The Efficacy and Safety of Probiotics for Prevention of Chemoradiotherapy‐Induced Diarrhea in People With Abdominal and Pelvic Cancer: A Systematic Review and Meta‐Analysis Based on 23 Randomized Studies.” International Journal of Surgery 84: 69–77. 10.1016/j.ijsu.2020.10.012 33080416

[15] Amiri Khosroshahi, Reza , Sheida Zeraattalab‐Motlagh , Peyman Sarsangi , Sabrina Mai Nielsen , and Hamed Mohammadi . 2023. “Effect of Probiotic Supplementation on Chemotherapy‐ and Radiotherapy‐Related Diarrhoea in Patients With Cancer: An Umbrella Review of Systematic Reviews and Meta‐Analyses.” British Journal of Nutrition 130: 1754–1765. 10.1017/s0007114523000910 37072129

[16] Carlson, Paul E . Jr. 2020. “Regulatory Considerations for Fecal Microbiota Transplantation Products.” Cell Host & Microbe 27: 173–175. 10.1016/j.chom.2020.01.018 32053787

[17] Huang, Bin , Mengxuan Gui , Honglin An , Jiayu Shen , Feimin Ye , Zhuona Ni , Hanzhang Zhan , et al 2023. “Babao Dan Alleviates Gut Immune and Microbiota Disorders While Impacting the TLR4/MyD88/NF‐кB Pathway to Attenuate 5‐Fluorouracil‐Induced Intestinal Injury.” Biomedicine & Pharmacotherapy 166: 115387. 10.1016/j.biopha.2023.115387 37643486

[18] Wang, Hao , Weize Zhu , Ying Hong , Wenjing Wei , Ningning Zheng , Xiaofang He , Yiyang Bao , et al. 2024. “Astragalus Polysaccharides Attenuate Chemotherapy‐Induced Immune Injury by Modulating Gut Microbiota and Polyunsaturated Fatty Acid Metabolism.” Phytomedicine 128: 155492. 10.1016/j.phymed.2024.155492 38479258

[19] Robertson, John M. , David Lockman , Di Yan , and Michelle Wallace . 2008. “The Dose‐Volume Relationship of Small Bowel Irradiation and Acute Grade 3 Diarrhea During Chemoradiotherapy for Rectal Cancer.” International Journal of Radiation Oncology, Biology, Physics 70: 413–418. 10.1016/j.ijrobp.2007.06.066 17904305

[20] Thomsen, Michael , and Luis Vitetta . 2018. “Adjunctive Treatments for the Prevention of Chemotherapy‐ and Radiotherapy‐Induced Mucositis.” Integrative Cancer Therapies 17: 1027–1047. 10.1177/1534735418794885 30136590 PMC6247548

[21] Bossi, P. , A. Antonuzzo , N. I. Cherny , O. Rosengarten , S. Pernot , F. Trippa , U. Schuler , et al. 2018. “Diarrhoea in Adult Cancer Patients: ESMO Clinical Practice Guidelines.” Annals of Oncology 29: iv126–iv142. 10.1093/annonc/mdy145 29931177

[22] Danne, Camille , Nathalie Rolhion , and Harry Sokol . 2021. “Recipient Factors in Faecal Microbiota Transplantation: One Stool Does Not Fit All.” Nature Reviews Gastroenterology & Hepatology 18: 503–513. 10.1038/s41575-021-00441-5 33907321

[23] Wei, Dang , Pauline Heus , Fleur T. van de Wetering , Geertjan van Tienhoven , Leen Verleye , and Rob Jpm Scholten . 2018. “Probiotics for the Prevention or Treatment of Chemotherapy‐ or Radiotherapy‐Related Diarrhoea in People With Cancer.” The Cochrane Database of Systematic Reviews 8: Cd008831. 10.1002/14651858.CD008831.pub3 30168576 PMC6513393

[24] Cani, Patrice D. , Clara Depommier , Muriel Derrien , Amandine Everard , and Willem M. de Vos . 2022. “ *Akkermansia muciniphila*: Paradigm for Next‐Generation Beneficial Microorganisms.” Nature Reviews. Gastroenterology & Hepatology 19: 625–637. 10.1038/s41575-022-00631-9 35641786

[25] Bian, Yingjie , Wei Li , Daniel M. Kremer , Peter Sajjakulnukit , Shasha Li , Joel Crespo , Zeribe C. Nwosu , et al. 2020. “Cancer *SLC43A2* Alters T Cell Methionine Metabolism and Histone Methylation.” Nature 585: 277–282. 10.1038/s41586-020-2682-1 32879489 PMC7486248

[26] Pandit, Mahesh , Yun‐Seo Kil , Jae‐Hee Ahn , Ram Hari Pokhrel , Ye Gu , Sunil Mishra , Youngjoo Han , et al. 2023. “Methionine Consumption by Cancer Cells Drives a Progressive Upregulation of PD‐1 Expression in CD4 T Cells.” Nature Communications 14: 2593. 10.1038/s41467-023-38316-9 PMC10162977 37147330

[27] Cole, Suzanne , Avneet Manghera , Lachrissa Burns , Janine Barrett , Nicole Yager , Hefin Rhys , Andrew Skelton , et al. 2023. “Differential Regulation of IL‐17A and IL‐17F via *STAT5* Contributes to Psoriatic Disease.” Journal of Allergy and Clinical Immunology 152: 783–798. 10.1016/j.jaci.2023.03.035 37244461

[28] Keir, Mary E. , Tangsheng Yi , Timothy T. Lu , and Nico Ghilardi . 2020. “The Role of IL‐22 in Intestinal Health and Disease.” The Journal of Experimental Medicine 217: e20192195. 10.1084/jem.20192195 32997932 PMC7062536

[29] Zhang, Peng , Junlai Liu , Allen Lee , Irene Tsaur , Masafumi Ohira , Vivian Duong , Nicholas Vo , et al. 2024. “IL‐22 Resolves MASLD via Enterocyte STAT3 Restoration of Diet‐Perturbed Intestinal Homeostasis.” Cell Metabolism 36: 2341–2354.e6. 10.1016/j.cmet.2024.08.012 39317186 PMC11631175

[30] Han, Chang , Caiping Shi , Linmeng Liu , Jichen Han , Qianqian Yang , Yan Wang , Xiaodan Li , et al. 2024. “Majorbio Cloud 2024: Update Single‐Cell and Multiomics Workflows.” iMeta 3: e217. 10.1002/imt2.217 39135689 PMC11316920

[31] Chen, Shifu , Yanqing Zhou , Yaru Chen , and Jia Gu . 2018. “Fastp: An Ultra‐Fast All‐in‐One FASTQ Preprocessor.” Bioinformatics 34: i884–i890. 10.1093/bioinformatics/bty560 30423086 PMC6129281

[32] Li, Heng , and Richard Durbin . 2009. “Fast and Accurate Short Read Alignment With Burrows–Wheeler Transform.” Bioinformatics 25: 1754–1760. 10.1093/bioinformatics/btp324 19451168 PMC2705234

[33] Li, Dinghua , Chi‐Man Liu , Ruibang Luo , Kunihiko Sadakane , and Tak‐Wah Lam . 2015. “MEGAHIT: An Ultra‐Fast Single‐Node Solution for Large and Complex Metagenomics Assembly via Succinct De Bruijn Graph.” Bioinformatics 31: 1674–1676. 10.1093/bioinformatics/btv033 25609793

[34] Fu, Limin , Beifang Niu , Zhengwei Zhu , Sitao Wu , and Weizhong Li . 2012. “Cd‐HIT: Accelerated for Clustering the Next‐Generation Sequencing Data.” Bioinformatics 28: 3150–3152. 10.1093/bioinformatics/bts565 23060610 PMC3516142

[35] Gu, Shengchang , Lin Fang , and Xun Xu . 2013. “Using SOAPaligner for Short Reads Alignment.” Current Protocols in Bioinformatics 44: 1–17. 10.1002/0471250953.bi1111s44 26270169

[36] Buchfink, Benjamin , Chao Xie , and Daniel H. Huson . 2015. “Fast and Sensitive Protein Alignment Using DIAMOND.” Nature Methods 12: 59–60. 10.1038/nmeth.3176 25402007

[37] Happel, Anna‐Ursula , Brian Kullin , Hoyam Gamieldien , Nicole Wentzel , Chambrez Z. Zauchenberger , Heather B. Jaspan , Smritee Dabee , et al. 2020. “Exploring Potential of Vaginal *Lactobacillus* Isolates From South African Women for Enhancing Treatment for Bacterial Vaginosis.” PLoS Pathogens 16: e1008559. 10.1371/journal.ppat.1008559 32497109 PMC7271994

[38] Koren, Sergey , Brian P. Walenz , Konstantin Berlin , Jason R. Miller , Nicholas H. Bergman , and Adam M. Phillippy . 2017. “Canu: Scalable and Accurate Long‐Read Assembly via Adaptive K‐Mer Weighting and Repeat Separation.” Genome Research 27: 722–736. 10.1101/gr.215087.116 28298431 PMC5411767

[39] Vaser, Robert , Ivan Sović , Niranjan Nagarajan , and Mile Šikić . 2017. “Fast and Accurate De Novo Genome Assembly From Long Uncorrected Reads.” Genome Research 27: 737–746. 10.1101/gr.214270.116 28100585 PMC5411768

[40] Hunt, Martin , Nishadi De Silva , Thomas D. Otto , Julian Parkhill , Jacqueline A. Keane , and Simon R. Harris . 2015. “Circlator: Automated Circularization of Genome Assemblies Using Long Sequencing Reads.” Genome Biology 16: 294. 10.1186/s13059-015-0849-0 26714481 PMC4699355

[41] Walker, Bruce J. , Thomas Abeel , Terrance Shea , Margaret Priest , Amr Abouelliel , Sharadha Sakthikumar , Christina A. Cuomo , et al. 2014. “Pilon: An Integrated Tool for Comprehensive Microbial Variant Detection and Genome Assembly Improvement.” PLoS One 9: e112963. 10.1371/journal.pone.0112963 25409509 PMC4237348

[42] Hyatt, Doug , Gwoliang Chen , Philip F. Locascio , Miriam L. Land , Frank W. Larimer , and Loren J. Hauser . 2010. “Prodigal: Prokaryotic Gene Recognition and Translation Initiation Site Identification.” BMC Bioinformatics 11: 119. 10.1186/1471-2105-11-119 20211023 PMC2848648

[43] Wu, Tianzhi , Erqiang Hu , Shuangbin Xu , Meijun Chen , Pingfan Guo , Zehan Dai , Tingze Feng , et al 2021. “Clusterprofiler 4.0: A Universal Enrichment Tool for Interpreting Omics Data.” Innovation 2: 100141. 10.1016/j.xinn.2021.100141 34557778 PMC8454663

[44] Zou, Yuanqiang , Wenbin Xue , Guangwen Luo , Ziqing Deng , Panpan Qin , Ruijin Guo , Haipeng Sun , et al. 2019. “1,520 Reference Genomes From Cultivated Human Gut Bacteria Enable Functional Microbiome Analyses.” Nature Biotechnology 37: 179–185. 10.1038/s41587-018-0008-8 PMC6784896 30718868

[45] Seemann, Torsten . 2014. “Prokka: Rapid Prokaryotic Genome Annotation.” Bioinformatics 30: 2068–2069. 10.1093/bioinformatics/btu153 24642063

[46] Xu, Heping , Jiarui Ding , Caroline B. M. Porter , Antonia Wallrapp , Marcin Tabaka , Sai Ma , Shujie Fu , et al. 2019. “Transcriptional Atlas of Intestinal Immune Cells Reveals That Neuropeptide α‐CGRP Modulates Group 2 Innate Lymphoid Cell Responses.” Immunity 51: 696–708.e9. 10.1016/j.immuni.2019.09.004 31618654 PMC6991097

[47] Hänzelmann, Sonja , Robert Castelo , and Justin Guinney . 2013. “GSVA: Gene Set Variation Analysis for Microarray and RNA‐seq Data.” BMC Bioinformatics 14: 7. 10.1186/1471-2105-14-7 23323831 PMC3618321

[48] Wu, Di , Zongxian Li , Yime Zhang , Yinlian Zhang , Guanqun Ren , Yanyu Zeng , Huiying Liu , et al. 2023. “Proline Uptake Promotes Activation of Lymphoid Tissue Inducer Cells to Maintain Gut Homeostasis.” Nature Metabolism 5: 1953–1968. 10.1038/s42255-023-00908-6 37857730

[49] Bolger, Anthony M. , Marc Lohse , and Bjoern Usadel . 2014. “Trimmomatic: A Flexible Trimmer for Illumina Sequence Data.” Bioinformatics 30: 2114–2120. 10.1093/bioinformatics/btu170 24695404 PMC4103590

[50] Edgar, Robert C . 2010. “Search and Clustering Orders of Magnitude Faster Than BLAST.” Bioinformatics 26: 2460–2461. 10.1093/bioinformatics/btq461 20709691

[51] Edgar, Robert C. , Brian J. Haas , Jose C. Clemente , Christopher Quince , and Rob Knight . 2011. “UCHIME Improves Sensitivity and Speed of Chimera Detection.” Bioinformatics 27: 2194–2200. 10.1093/bioinformatics/btr381 21700674 PMC3150044

[52] Callahan, Benjamin J. , Paul J. McMurdie , Michael J. Rosen , Andrew W. Han , Amy Jo A. Johnson , and Susan P. Holmes . 2016. “DADA2: High‐Resolution Sample Inference From Illumina Amplicon Data.” Nature Methods 13: 581–583. 10.1038/nmeth.3869 27214047 PMC4927377

[53] Quast, Christian , Elmar Pruesse , Pelin Yilmaz , Jan Gerken , Timmy Schweer , Pablo Yarza , Jörg Peplies , and Frank Oliver Glöckner . 2012. “The SILVA Ribosomal RNA Gene Database Project: Improved Data Processing and Web‐Based Tools.” Nucleic Acids Research 41: D590–D596. 10.1093/nar/gks1219 23193283 PMC3531112

