# Supplementary material for: Gut‐derived Lactobacillus from exceptional responders mitigates chemoradiotherapy‐induced intestinal injury through methionine‐driven epigenetic modulation
Source: Imeta. 2025 May 14;4(3):e70043. doi: 10.1002/imt2.70043 (PMC12130557; doi:10.1002/imt2.70043)
Supplement: Supplementary file 1 — Figure S1. Gut microbiota influences acute chemoradiotherapy‐induced intestinal injury (ACRIII). Figure S2. Screening of Lactobacillus strains derived from exceptional rectal cancer patients. Figure S3. Safety of Lacticaseibacillus rhamnosus (L. rhamnosus) DY801. Figure S4. Exploration of the single and combined effect of novel strains from exceptional patients in alleviating ACRIII. Figure S5. L. rhamnosus DY801 inhibits inflammation and enhances tumor therapy for subcutaneous tumors. Figure S6. Pan‐genomic analysis and function of the metB gene. Figure S7. In vitro and in vivo experiments with lymphoid tissue inducer (Lti) cells. Figure S8. Cut & Tag sequencing of Lti cells treated with methionine or knockdown of genes. Figure S9. In vivo experiments validate that S‐adenosylmethionine (SAM) affects the length of jejunal villi and cyrpt. [file IMT2-4-e70043-s002.docx]

Supporting information to

Gut-Derived *Lactobacillus* from Exceptional Responders Mitigates Chemoradiotherapy-Induced Intestinal Injury through Methionine-Driven Epigenetic Modulation

**Running title:** New *Lactobacillus* strains alleviate ACRIII.

Lu Yu^1#^, Zhenhui Chen^2#^, Shengqi Yin^1^, Qiqing Guo^1^, Yuchuan Chen^3^, Jiaying Li^1^, Yafang Wang^1,4^, Xiangqiang Liu^5^, Zi Xu^1^, Yaowei Zhang^1^, Yuqin Zhang^1^, Zhihao Zheng^1^, Keli Chen^6^, Yanqing Ding^7^, Hongying Fan^2*^, Zhifeng Liu^8*^, Yi Ding^1*^

^1^Department of Radiation Oncology, Nanfang Hospital, Southern Medical University, Guangzhou 510515, China

^2^Department of Microbiology, Guangdong Provincial Key Laboratory of Tropical Disease Research, School of Public Health, Southern Medical University, Guangzhou 510515, China

^3^State Key Laboratory of Organ Failure Research, Key Laboratory of Infectious Diseases Research in South China, Ministry of Education, Guangdong Provincial Key Laboratory of Viral Hepatitis Research, Guangdong Provincial Clinical Research Center for Viral Hepatitis, Department of Infectious Diseases, Nanfang Hospital, Southern Medical University, Guangzhou 510515, China

^4^Department of Radiotherapy, General Hospital of Southern Theatre Command, Guangzhou 510515, China

^5^Department of Gastroenterology, General Hospital of Southern Theatre Command, Guangzhou 510515, China

^6^HuiQiao Medical Center, Nanfang Hospital, Southern Medical University, Guangzhou 510515, China

^7^Guangdong Province Key Laboratory of Molecular Tumor Pathology, Guangzhou 510515, China.

^8^The Department of Critical Care Medicine, General Hospital of Southern Theater Command of PLA, Guangzhou 510010, China

^#^These authors contributed equally: Lu Yu, Zhenhui Chen.

*Correspondence: [dy512@smu.edu.cn](mailto:dy512@smu.edu.cn) (Yi Ding), [Zhifengliu7797@163.com](mailto:Zhifengliu7797@163.com) (Zhifeng Liu), [gzfhy@smu.edu.cn](mailto:gzfhy@smu.edu.cn) (Hongying Fan)


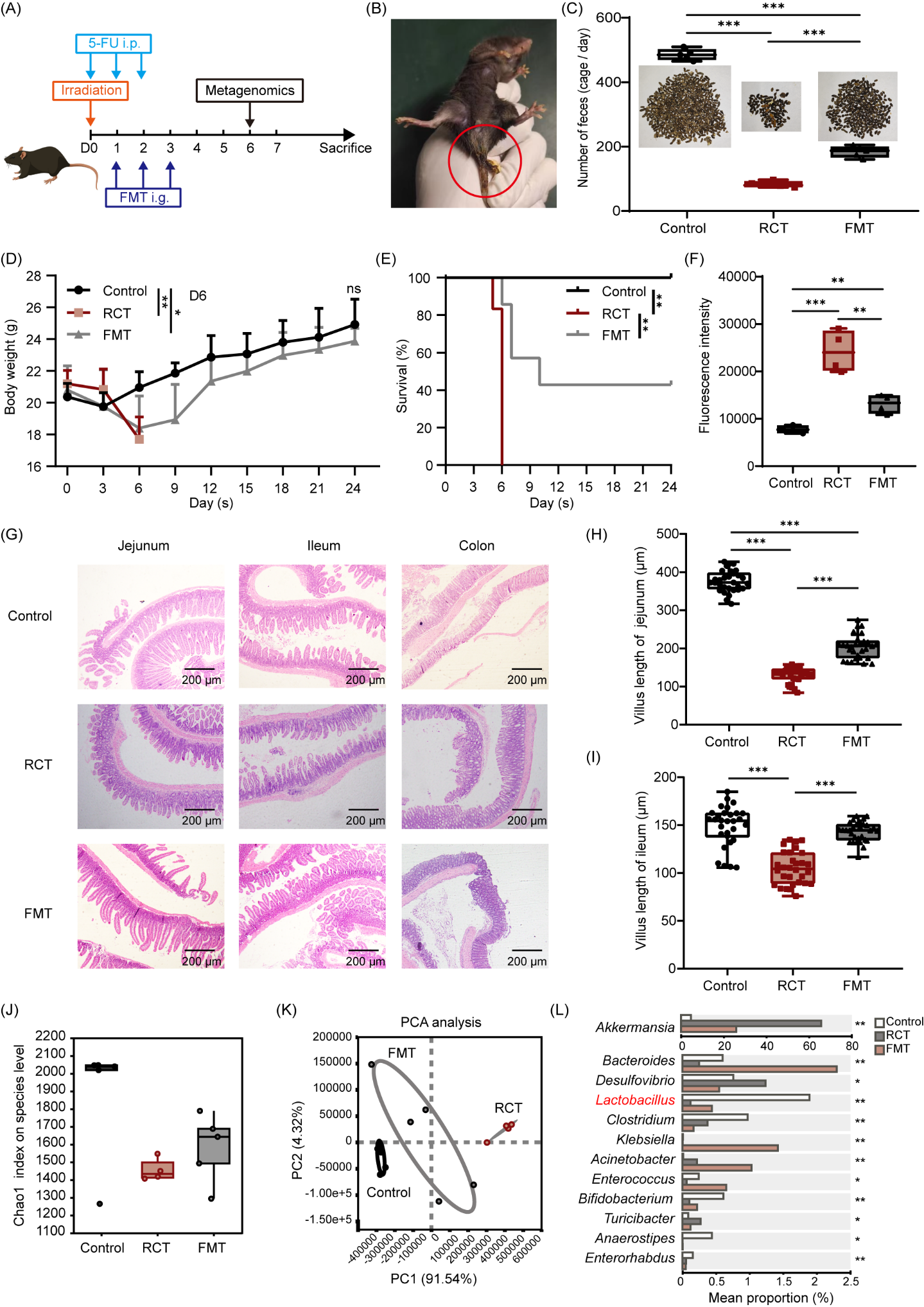


Figure S1 Gut microbiota influences acute chemoradiotherapy-induced intestinal injury (ACRIII). (A) Schematic representation of facal microbiota transplantationg (FMT), ACRIII induction, and cecal content collection for metagenomics. (B) Signs of diarrhea in mice with feces on their tails. (C) Box plots showing the counts of formed feces in each group. (D) The body weight changes in mice after FMT and ACRIII induction. *n* = 7 mice per group. Statistical analysis of body weight on Day 6. (E) The survival rate in mice. (F) Box plots displaying the fluorescein isothiocyanate (FITC) fluorescence intensity in the blood of mice from different groups. (G) Representative HE images in the jejunum, ileum, and colon. (H) Box plots showing quantification of villus length in the jejunum. (I) Box plots showing quantification of villus length in the ileum. (J) Box plot comparing the difference of alpha diversity (Chao1) in each group. (K) Principal component analysis (PCA) plot showing the difference of beta diversity in each group. (L) Bar chart showing differential bacterial genus in each group. Significance: * *p* < 0.05, ** *p* < 0.01, and *** *p* < 0.001; data are representative of two or three independent experiments. 5-Fu, 5-fluorouracil; RCT, radiochemotherapy; FMT, fecal microbiota transplantation; i.p., intraperitoneal; i.g., intragastric; FITC, fluorescein isothiocyanate; PCA, principal component analysis; HE, hematoxylin and eosin staining.

**
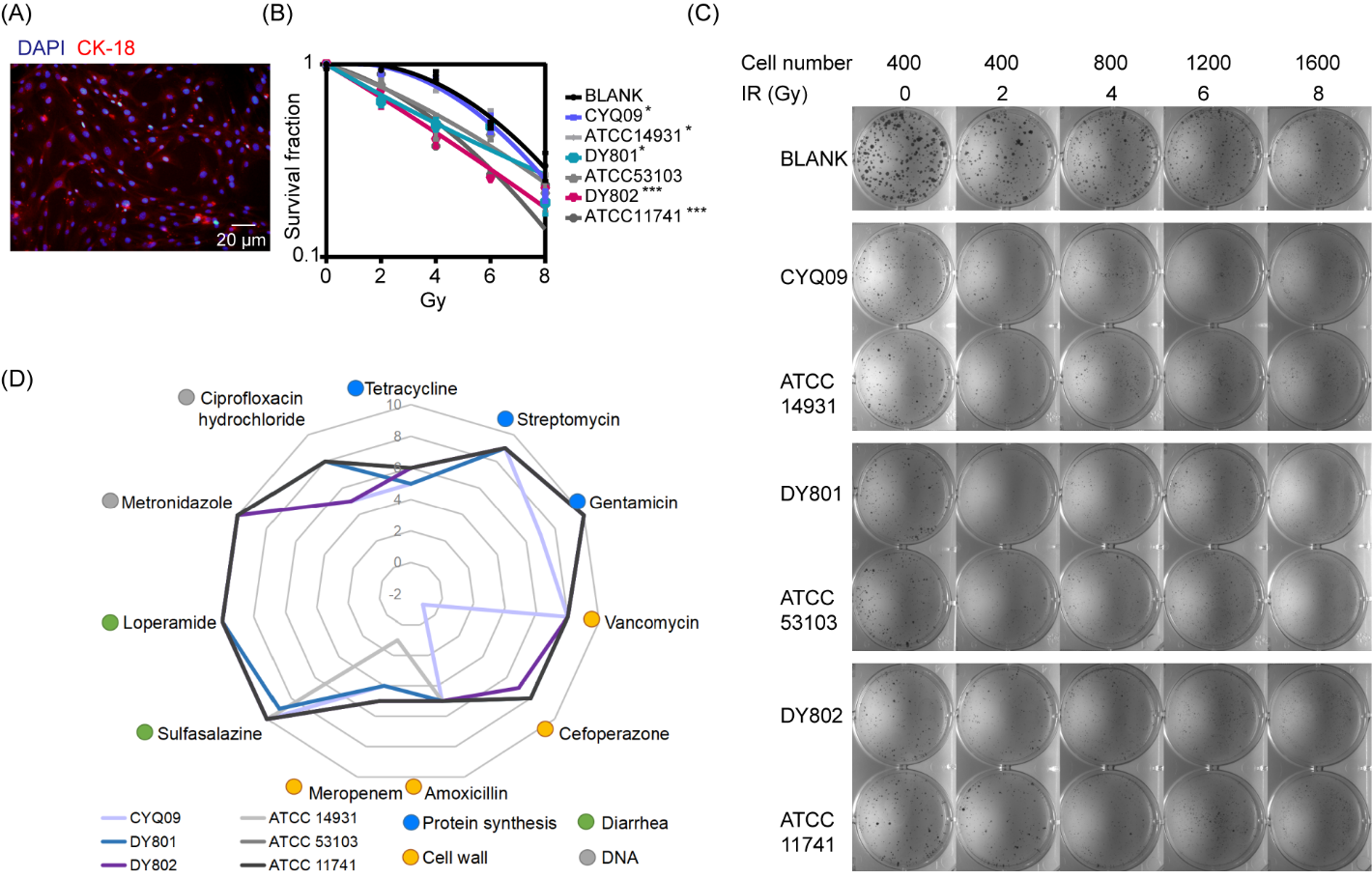
**

Figure S2 Screening of *Lactobacillus* strains derived from exceptional rectal cancer patients. (A) Representative immunofluorescence images showing cytokeratin-18 (CK-18) in red and 4',6-diamidino-2-phenylindole (DAPI) in blue. (B) Fitted curves demonstrating the radioresistance for MC38 cells treated with bacterial supernatants from *Lactobacillus* strains. (C) Representative images of colony assay for MC38 cells treated with bacterial supernatants from *Lactobacillus* strains. (D) Radar plots showing the minimal inhibitory concentration (MIC) of each bacterial strain under different drug types, with MIC values log2-transformed. Significance: * *p* < 0.05, ** *p* < 0.01, and *** *p* < 0.001; data are representative of two or three independent experiments. DAPI, 4',6-diamidino-2-phenylindole; MIC, minimal inhibitory concentration.

**
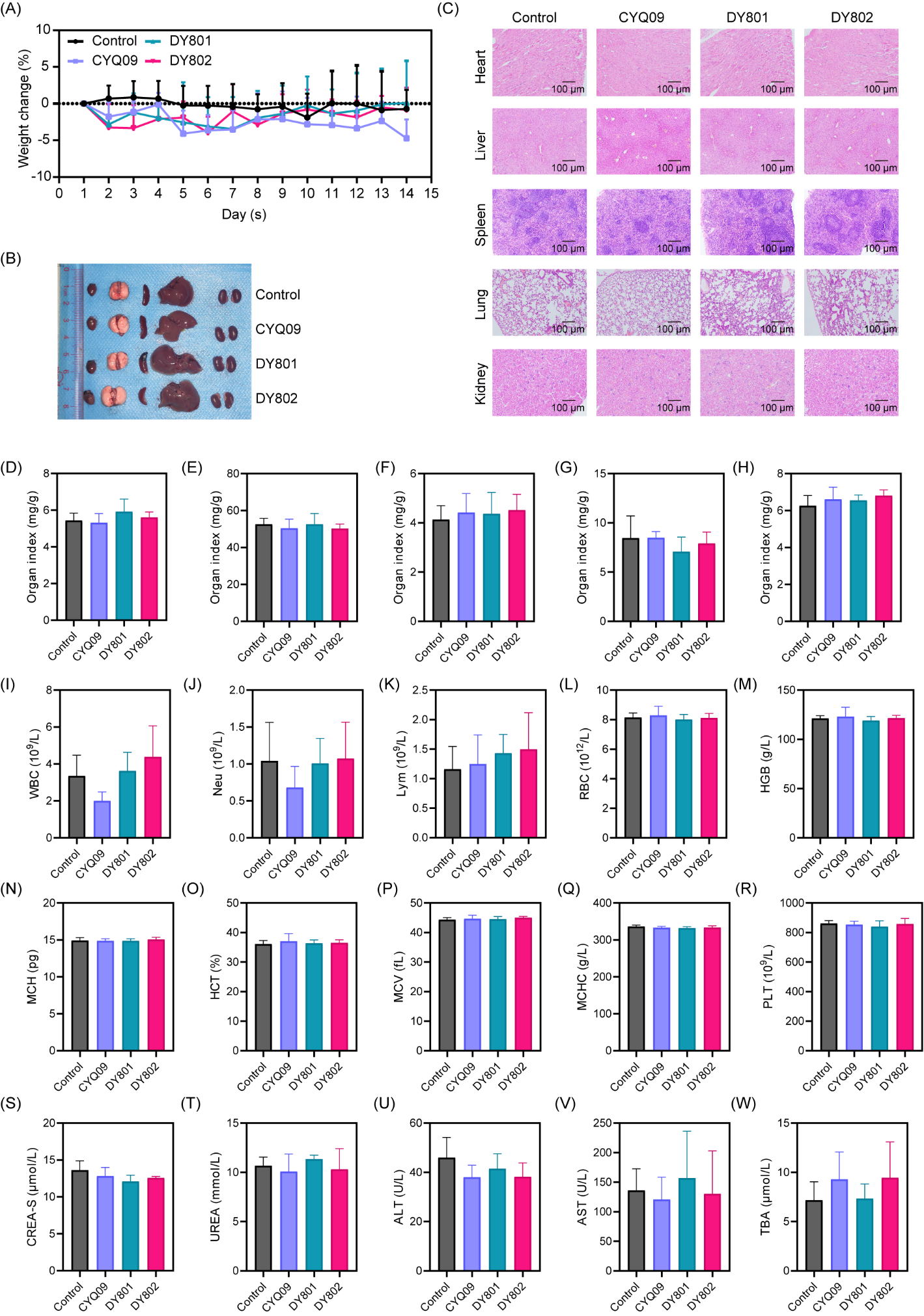
**

Figure S3 Safety of *Lacticaseibacillus rhamnosus* (*L. rhamnosus*) DY801. (A) The body weight changes in mice after high-dose gavage of DY801. (B) Representative images of vital organs in mice. (C) Representative HE images in the vital organs. (D-H) Bar plots showing the organ indices of heart (D), liver (E), spleen (F), lung (G) and kidney (H) in mice from different group. (I-L) Bar plots showing the counts of white blood cells (I), neutrophil cells (J), lymphocyte cells (K), and red blood cells (L) in blood from different mouse groups. (M-Q) Bar plots showing the concentration of hemoglobin (M), mean corpuscular hemoglobin (N), percentage of hematocrit (O), mean corpuscular volume (P), and mean hemoglobin concentration (Q) in blood from different mouse groups. (R) Bar plots showing the counts of platelets in blood from different mouse groups. (S-W) Bar plots showing the concentration of creatinine (S), urea (T), alanine transferase (U), aspartate transferase (V), and total bile acids (W) in blood from different mouse groups. Data are representative of two or three independent experiments. WBC, white blood cells; Neu, neutrophil cells; Lymt, lymphocyte cells; RBC, red blood cell; HGB, hemoglobin; MCH, mean corpuscular hemoglobin; HCT, hematocrit; MCV, mean corpusular volume; MCHC, mean hemoglobin concentration; PLT, Platelet; CREA-S, Creatinine; ALT, alanine transferase; AST, aspartate transferase; TBA, total bile acids; HE, hematoxylin and eosin staining.


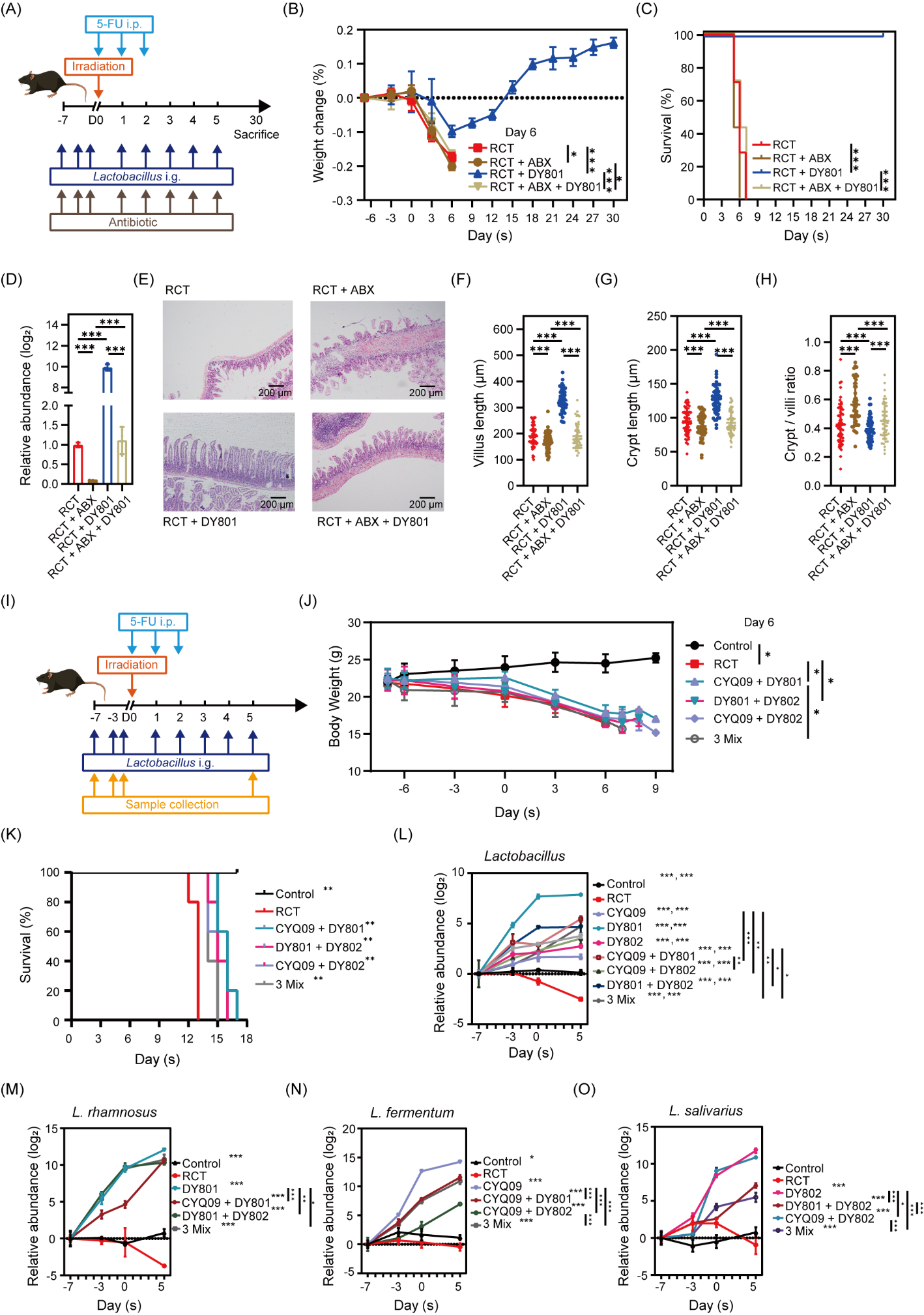


Figure S4 Exploration of the single and combined effect of novel strains from exceptional patients in alleviating ACRIII. (A) Schematic representation of ampicillin treatment, DY801 gavage and ACRIII induction. (B and C) Body weight changes (B) and survival rates (C) of mice after antibiotic treatment, DY801 gavage and ACRIII induction. *n* = 7 mice per group. (D) Relative abundance of *L. rhamnosus* in intestinal content at Day 6, with values log2-transformed. (E) Representative HE images in the jejunum from each group. (F) Box plots showing quantification of villi length in the jejunum. (G) Box plots showing quantification of crypt length in the jejunum. (H) Box plots showing quantification of crypt / villus ratio in the jejunum. (I-O) The exploration of the combined effect of novel strains from exceptional patients in alleviating ACRIII. (I) Schematic of *Lactobacillu*s multi-colonization in ACRIII mice model. (J) The body weight changes and (K) the survival rate in mice after *Lactobacillus* gavage and ACRIII induction. *n* = 5 mice per group. Statistical analysis of body weight on Day 6. (L) The relative abundance of the *Lactobacillus* genus in feces at different time points was measured with qPCR. The data was normalized using log2 change. (M) The relative abundance of the *L. rhamnosus*. (N) The relative abundance of the *L. fermentum*. (O) The relative abundance of the *L. salivarius*. Stars in the upper right corners of each panel in (L)-(O) denote comparisons with the RCT group for statistical analysis. Significance: * *p* < 0.05, ** *p* < 0.01, and *** *p* < 0.001; data are representative of two or three independent experiments. ABX, antibiotic; 3 Mix, a mixture of *Lactobacillus* CYQ09, DY801 and DY802; qPCR, quantitative real-time polymerase chain reaction‌‌.

**
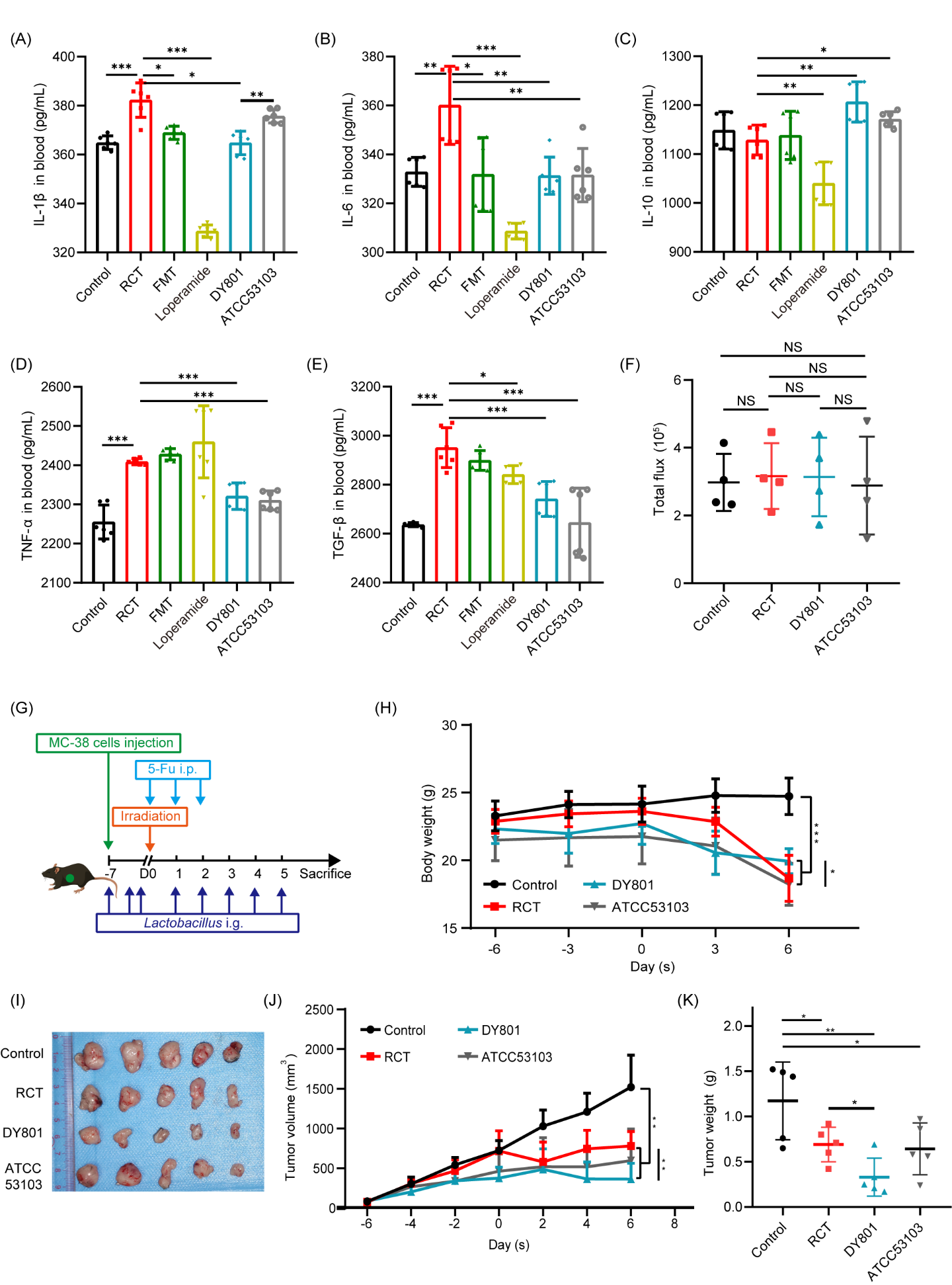
**

Figure S5 *L. rhamnosus* DY801 inhibits inflammation and enhances tumor therapy for subcutaneous tumors. (A) The interleukin-1 beta (IL-1β) level in the blood of mice in each group. (B) The interleukin-6 (IL-6) level in each group. (C) The interleukin-10 (IL-10) level in each group. (D) The tumor necrosis factor-alpha (TNF-α) level in each group. (E) The transforming growth factor-beta (TGF-β) level in each group. (F) The total flux intensity of tumor models in situ tumor model. (G) Schematic of *Lactobacillus* monocolonization, MC38 cell injection and ACRIII induction. (H) The body weight changes and in mice after bacteria monocolonization, MC38 injection and ACRIII induction. *n* = 5 mice per group. Statistical analysis of body weight on Day 6. (I) Representative images of tumors. (J) The tumor volume changes and in mice after bacteria monocolonization, MC38 injection and ACRIII. (K) Dot plot showing the tumor weight of each group. Significance: NS, *p* > 0.05, * *p* < 0.05, ** *p* < 0.01, and *** *p* < 0.001; data are representative of two or three independent experiments.

**
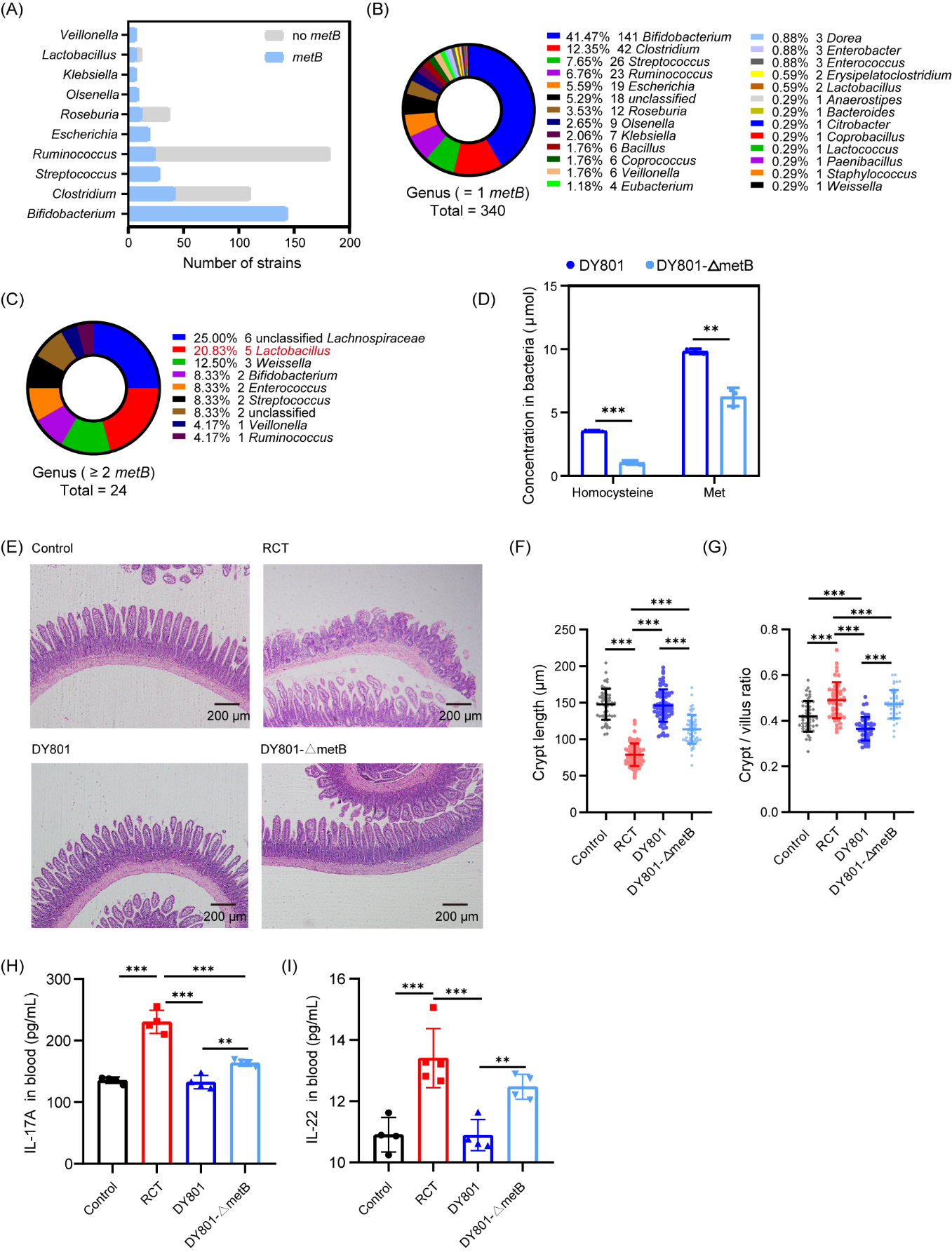
**

Figure S6 Pan-genomic analysis and function of the *metB* gene. (A) Top ten genus containing *metB* genes in human gut microbiota. (B) Composition of genus containing 1 metB gene. (C) Composition of genera containing 2 and more *metB* genes. (D) Bar plot shows liquid chromatography–mass spectrometry (LC-MS) quantification of homocysteine and methionine in the bacteria cytoplasm from DY801 and DY801-△metB strains cultured in medium supplemented with 0.5% L-cysteine for 24 h. Homocysteine and methionine abundances are indicated by the peak area in the mass spectrum. (E) Representative HE images in the jejunum from each group. (F) Box plots showing quantification of crypt length in the jejunum. (G) Box plots showing quantification of crypt / villus ratio in the jejunum. (H) Bar charts show interleukin-17A (IL-17A) level in the blood of mice from each group. (I) Bar charts show interleukin-22 (IL-22) level in the blood of mice from each group. Significance: ** *p* < 0.01, and *** *p* < 0.001; data are representative of two or three independent experiments.

**
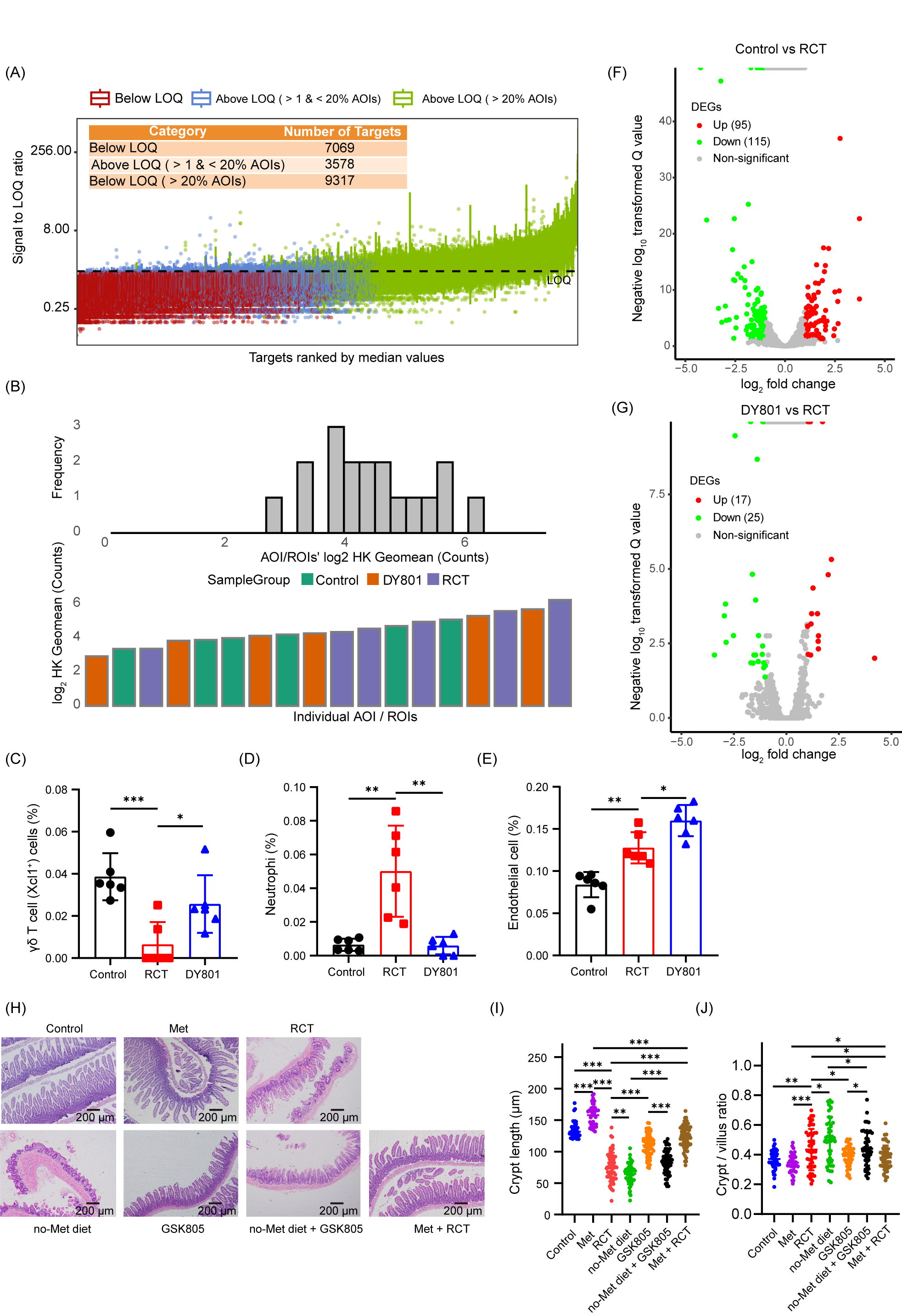
**

Figure S7 *In vitro* and *in vivo* experiments with lymphoid tissue inducer (Lti) cells. (A) Scatter plot showing the distribution of target signal ratio relative to the limit of quantification (LOQ), with the y-axis log2-transformed. (B) Bar graph showing the expression distribution of housekeeping genes, with geometric mean log2-transformed across the dataset. (C) Bar graph showing the proportion of γδ T cells within each region of interest (ROI) of immune cells. (D) Bar graph showing the proportion of neutrophi within each ROI of immune cells. (E) Bar graph showing the proportion of endothelial cells within each ROI of immune cells. (F) Volcano plot illustrating differentially expressed genes (DEGs) between Control and RCT groups in Lti cells. (G) Volcano plot illustrating DEGs between DY801 and RCT groups in Lti cells. (H) Representative HE images in the jejunum from each group. (I) Box plots showing quantification of crypt length in the jejunum. (J) Box plots showing quantification of crypt / villus ratio in the jejunum. Significance: * *p* < 0.05, ** *p* < 0.01, and *** *p* < 0.001; data are representative of two or three independent experiments. LOQ, limit of quantification; Met, methionine; no-Met diet, methionine-deficient diet; ROI, region of interest; DEGs, differentially expressed genes.


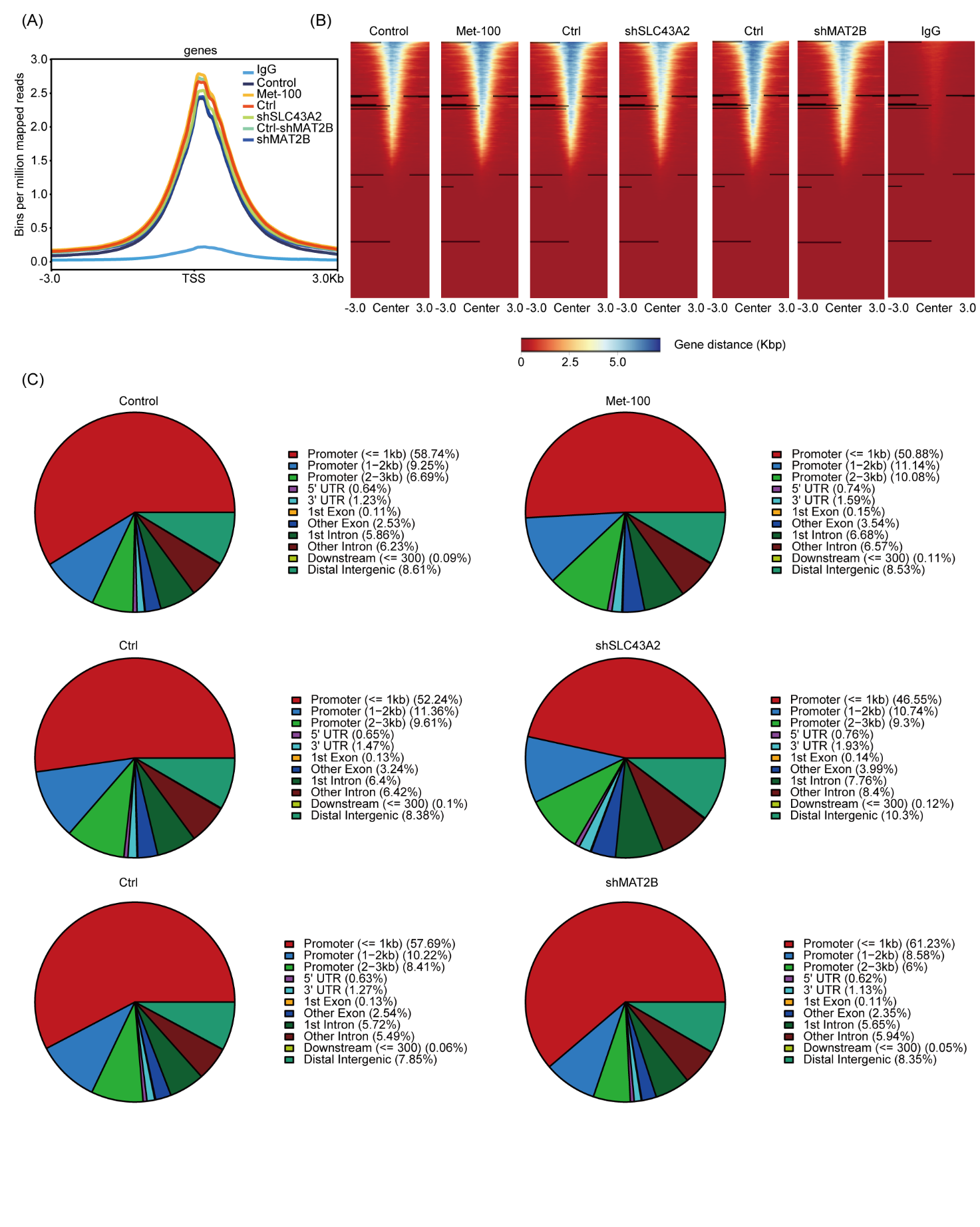


Figure S8 Cut & Tag sequencing of Lti cells treated with methionine or knockdown of genes. (A) Line graph of signal distribution around transcription start site (TSS). (B) Heatmap of signal distribution around TSS. (C) pie chart of peak distribution across gene elements. Met-100, 100 mM of methionine; TSS, transcription start site.


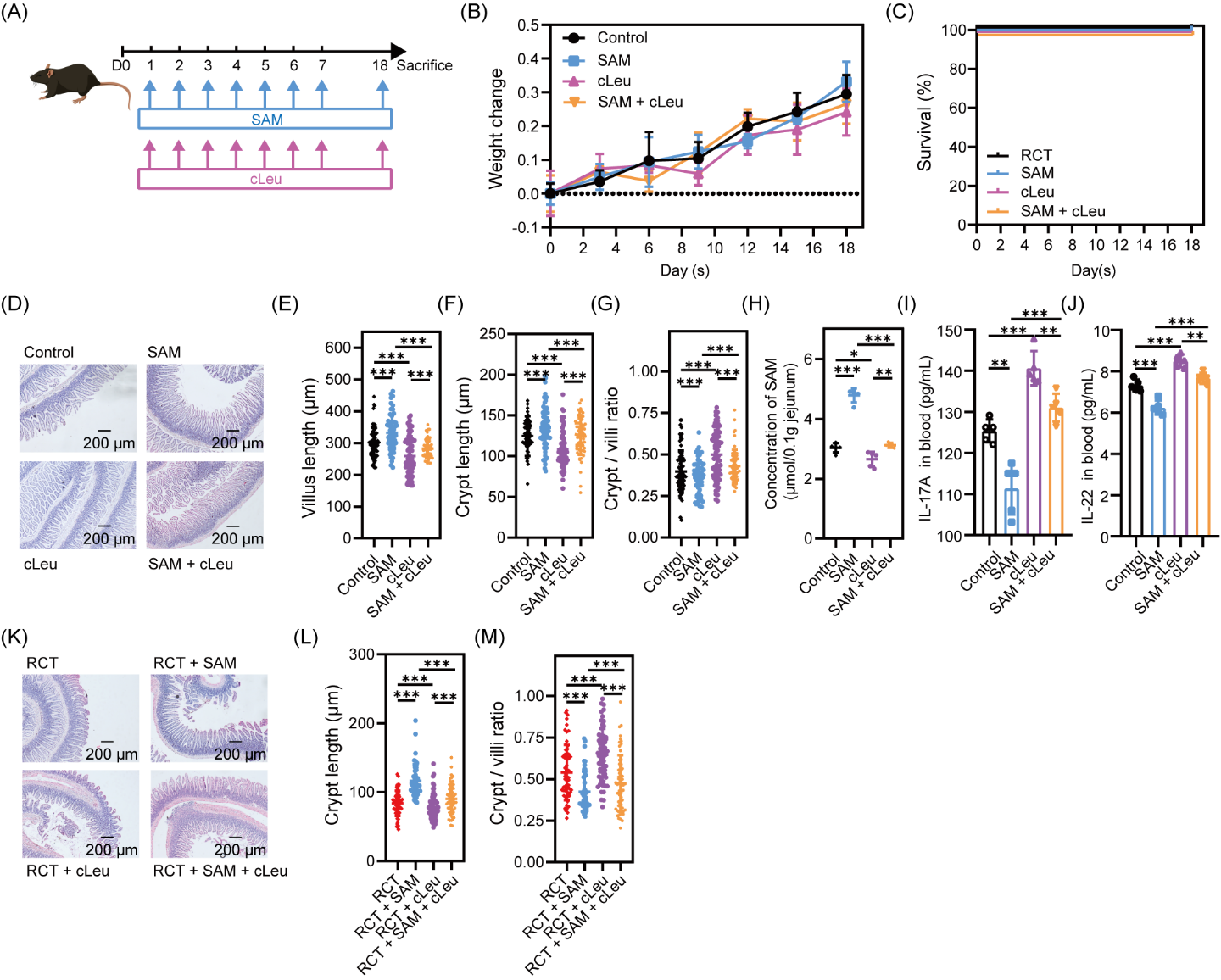


Figure S9 *In vivo* experiments validate that **S-adenosylmethionine** (SAM) affects the length of jejunal villi and cyrpt. (A) Schematic representation of cycloleucine (cLeu) and SAM treatment. (B) Body weight changes of mice after metabolites treatment and ACRIII induction. *n* = 7 mice per group. (C) Survival rates of mice. (D) Representative HE images in the jejunum from each group. (E) Box plots showing quantification of villi length in the jejunum. (F) Box plots showing quantification of crypt length in the jejunum. (G) Box plots showing quantification of crypt / villus ratio in the jejunum. (H) Scatter plot showing LC-MS quantification of SAM in jejunum harvested from the mice in each group. SAM abundance was indicated by the peak area in the mass spectrum. (I) Bar charts show IL-17A level in the blood of the mice in each group. (J) Bar charts show IL-22 level in the blood of the mice in each group. (K) Representative HE images in the jejunum from each group. (L) Box plots showing quantification of crypt length in the jejunum. (M) Box plots showing quantification of crypt / villus ratio in the jejunum. Significance: * *p* < 0.05, ** *p* < 0.01, and *** *p* < 0.001; data are representative of two or three independent experiments.cLeu, cycloleucine.
